# Supplementary material for: The influence of nonlinear resonance on human cortical oscillations
Source: Commun Biol. 2026 May 4;9:605. doi: 10.1038/s42003-026-10164-5 (PMC13144324; doi:10.1038/s42003-026-10164-5)
Supplement: Supplementary file 2 — Supplementary Information [file 42003_2026_10164_MOESM2_ESM.pdf]

Supplementary Materials

S.1 Dataset, Analysis-Parameter, and Notation Tables

Table S 1: iEEG Dataset Description (Frauscher et al., 2018)[1]

Parameter	Description
Dataset name	MNI Open iEEG Atlas
Reference	Frauscher, B., et al. (2018). Atlas of the normal intracranial electroencephalogram. Brain, 141(4), 1130-1144
Data repository	https://mni-open-ieegatlas.research.mcgill.ca/
Subjects	106 patients with therapy-refractory focal epilepsy
Demographics	54 males; mean age 33.1 ± 10.8 years
Electrode types	SEEG (stereo-EEG): 89 patients (84%), 1,520 channels; ECoG (grids/strips): 17 patients (16%), 265 channels
Total channels	1,785 bipolar channels (left hemisphere: 1,066; right hemisphere: 719)
Channel definition	Bipolar derivation
Recording condition	Resting wakefulness, eyes closed
Epoch duration	60-second artifact-free sections

Table S 2: Scalp EEG Dataset Description (Li et al., 2022)[2]

Parameter	Description
Dataset name	HarMNqEEG (Harmonized Multinational qEEG Norms)
Reference	Li, M., et al. (2022). Harmonized-Multinational qEEG Norms. *NeuroImage*, 256, 119190
Data repository	https://www.synapse.org/HarMNqEEG (Synapse ID: syn26712693)
Subjects	1,564 healthy participants (783 females, 781 males)
Age range	5-95 years (lifespan dataset)

Countries	9 countries: Barbados, China, Colombia, Cuba, Germany, Malaysia, Russia, Switzerland, USA
Recording devices	12 different EEG devices across sites
Channels	19 channels (10-20 International System: Fp1, Fp2, F3, F4, C3, C4, P3, P4, O1, O2, F7, F8, T3/T7, T4/T8, T5/P7, T6/P8, Fz, Cz, Pz)
Reference	Average reference; Pz electrode kept in this paper since it is univariate analysis
Recording condition	Resting-state, eyes closed, quasi-stationary
Epoch duration	>= 1 minute artifact-free; segmented into 2.56 s epochs (frequency resolution: 0.39 Hz)
Frequency range	Amplifiers: 0.5-35 Hz; analysis restricted to 1.17-19.14 Hz
Preprocessing	(1) Within-site artifact rejection; (2) Average re-referencing; (3) Maximum likelihood shrinkage for positive-definite cross-spectra; (4) Global scale factor (GSF) correction
Quality control	Three-stage: local site filtering, neurophysiologist visual inspection, machine-learning outlier detection (t-SNE + robust Mahalanobis distance)
Outlier exclusion	191 subjects identified as outliers and excluded from normative analysis
Our subset	960 subjects retained (excluded subjects with <140 usable segments to match iEEG segment count of 157)

Table S 3: Our Analysis Parameters (Both Datasets)

Parameter	Description
Sampling rate	200 Hz
Window length	1.5 s (300 samples)
FFT length	300
Overlap	75%
Frequency range	1.5-45 Hz
Spectral method	Multitaper (pmtm) with sine tapers
Time-bandwidth product	$NW = 1.5$ ($K = 2NW-1 = 2$ tapers)
Bicoherence normalization	Haubrich (1965)

Segments per channel	157 (matched across datasets)
iEEG montage	Bipolar (original atlas format)
Scalp EEG montage	Average reference (19 channels)

Table S 4 Summary of symbols

**Symbol**	**Definition**
$x \in \mathbb{C}$ or $x \in \mathbb{C}$	Scalar
$\boldsymbol{x} \in \mathbb{C}^{d}$	Vector of size d (e.g., $\boldsymbol{x} = [x_i \| i = 1, \cdots, d]$)
$\boldsymbol{X} \in \mathbb{C}^{d_1 \times d_2}$	Matrix of size $d_1 \times d_2$
i	Index of individual $i \in \{1, 2, \cdots N_i\}$, N_i is the total participants or subjects in the study
c	Index of the channel $c \in \{1, 2, \cdots, N_c\}$, N_c is total EEG channels
e	Index of EEG epochs $e \in \{1, 2, \cdots, N_e\}$, N_e is the disjoint segments selected from the continuous EEG
f	Physical frequencies with unit Hz
t	Physical time of each epoch
k	Index of taper, $k \in \{1, 2, \cdots, N_k\}$, where $N_k = 2NW - 1$
NW	Time-bandwidth product. N is the number of time samples in a single EEG epoch. W is the half-bandwidth of the tapers

Table S 5 Symbol of quantities and operators

**Symbol**	Name	**Definition**
$v_{i,c,e}(t) \in \mathbb{R}$<br>$v_{i,c,e}(f) \in \mathbb{C}$	EEG potential and its Discrete Fourier transform	EEG potential of the individual i, channel c of e-th epoch at a time t or frequency f for the Discrete Fourier transform.
$S_{i,c}(f) \in \mathbb{R}^{+}$	Theoretical spectra	The theoretical spectrum of an individual i channel c at the frequency f

$\hat{S}_{i,c}(f) \in \mathbb{R}^+$	Power spectra	The empirical variance vector of $v_{i,c,e}(f)$ across all epochs for an individual i at the frequency f
$B_{i,c}(f_1, f_2) \in \mathbb{C}$	Theoretical Bispectrum	The theoretical bispectrum of an individual channel c at the bifrequency (f_1, f_2)
$\widehat{B}_{i,c}(f_1, f_2) \in \mathbb{C}$	Empirical Bispectrum	The empirical bispectrum of process $v_{i,c,e}(t)$ across all epochs for an individual i channel c at the bifrequency (f_1, f_2)
$b_{i,c}(f_1, f_2) \in \mathbb{C}$	Population Bicoherence	Population normalized bispectrum for individual i and channel c at the bifrequency (f_1, f_2)
$\widehat{b}_{i,c}(f_1, f_2) \in \mathbb{C}$	Multitaper Bicoherence	Multitaper bicoherence estimator for individual i and channel c for bifrequency (f_1, f_2)
$\tilde{b}_{i,c}(f_1, f_2) \in \mathbb{C}$	BisCA Bicoherence	BiSCA -model estimator for individual i and channel c for bifrequency (f_1, f_2)
$F_s \in \mathbb{R}^+$	Sampling frequency	Sampling frequency in Hz. Common sampling rate after resampling for all individuals and channels
$U_2(i, c) \in \mathbb{R}$	Second-order cumulant	Second-order cumulant statistic of the empirical bicoherence distribution for individual i, channel c
$U_3(i, c) \in \mathbb{R}$	Third-order cumulant	Third-order cumulant statistic of the empirical bicoherence distribution for individual i, channel c
$k_i \in \{1, 2, \cdots, K\}$	Taper index	Index of taper, where K is the number of orthogonal tapers
$b_{i,c}^{(G)} \in \mathbb{R}^+$	Gaussianity test statistic	Median bicoherence magnitude across the bifrequency domain for individual i, channel c. Used to test for non-Gaussianity
$b_{i,c}^{(L)} \in \mathbb{R}^+$	Linearity test statistic	Maximum bicoherence magnitude across the bifrequency domain for individual i, channel c. Used to test for nonlinearity
$c_{\alpha}^{(G)} \in \mathbb{R}^+$	Gaussianity critical value	Critical value for the Gaussianity test at significance level α, derived from the null distribution

$c_{\alpha}^{(L)} \in \mathbb{R}^{+}$	Linearity critical value	Critical value for the linearity test at significance level α, derived from the Gumbel extreme-value distribution
$N_s^{eff}(i,c) \in \mathbb{Z}^{+}$	Effective segment count	Effective number of independent segments for individual i, channel c, after accounting for overlap and tapering
$\mathrm{E}[\cdot]$	Expectation	The expectation of a random variable
$\mathrm{Var}[\cdot]$	Variance	The variance of a random variable

S.2 Summary of EEG Spectral Parameterization Methods

As a typical machine learning problem, many toolboxes implemented the curve fitting for spectroscopic data analysis — such as SpectroChemPy [3], PNNL Chemometric Toolbox[4] astronomy data modeling [5], SLM (Shape Language Modeling) [6], LMFIT (Levenberg-Marquardt least-squares minimization) [7]. This paper emphasizes studies that have been applied specifically to EEG spectral analysis with certain constraints of biophysical prior.

Table S 6 Summary of EEG Spectral Parameterization Methods

Method	Feature
SPA (Spectral Parameter Analysis)[8–10]	Calculate the resonance peaks information from the poles and zeros of the autoregressive model
Xi-Alpha (ξα)[11]	Additive t-student kernels in nature scale fitted with maximum Whittle's likelihood
BOSC (Better Oscillation Detection)[12–14]	Thresholds for time and amplitude to detect and parametrize the burst of rhythm in time frequency
Gaussian peak fit[15]	Least square Gaussian peak fit
IRASA (Irregular-Resampling Auto-Spectral Analysis)[16]	Resampling to shift the spectra and using a media curve to get components
SpecParam (Spectral parameterization) or FOOOF (Fitting Oscillations & One Over F)[17]	Sequentially fit peaks through least square Gaussian fit in the log-scale
SPRiNT (Spectral Parameterization Resolved in Time)[18]	Parametrize the peaks in the smoothed short-time Fourier transforms
PaWNextra (Pink and White Noise extraction)[19]	Least square fit
Cortical Xi-Alpha[20]	Nonnegative matrix decomposition for multichannel

Xi-Pi (ξ-π)[21]	Fit nonparametric spectrum components with Whittle's likelihood
Xi-Alpha Net[22]	Hierarchical Bayesian model for solving the State space model with Xi-Alpha prior to reconstruct the source space spectrum
Our BiSCA (Higher-order Xi-Alpha Model)	Parametrize both spectrum and bispectrum with joint likelihood

S.3 Simulation

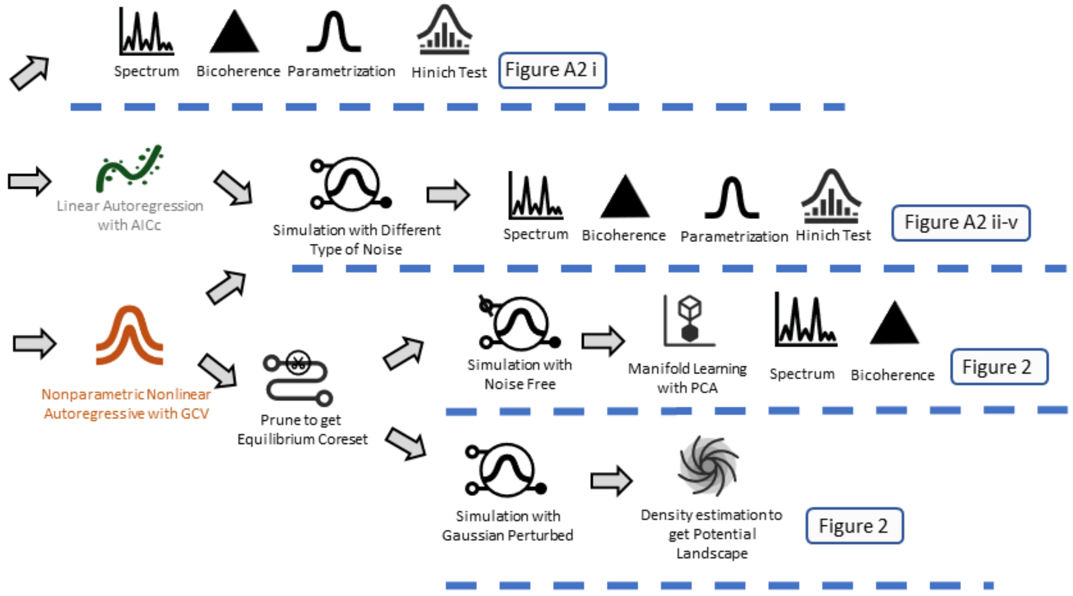

Fig. S 1. Flowchart of the Simulation and Analysis Pipeline. *This diagram illustrates the methodology for analyzing real intracranial EEG (iEEG) data and generating four corresponding types of synthetic surrogate data to test for nonlinearity and non-Gaussianity. (1) Direct Analysis of Real Data (Top Path, Ref: Fig. S2 i): The process begins with a real iEEG time series. This signal is directly subjected to spectral and bispectral analysis to compute its empirical power spectrum and bicoherence as a real-data baseline. (2) Linear and Nonlinear Surrogate Generation (Middle Path, Ref: Fig.S2 ii-v): A linear Autoregressive (AR) and a Nonparametric Nonlinear Autoregressive (NNAR) model is fitted to the real iEEG data. The optimal model order is selected using the Akaike Information Criterion corrected (AICc). This AR model and NNAR model are then used to generate two types of time series by driving it with different stochastic innovations: Linear/Nonlinear Gaussian: Using Gaussian (normal) distributed noise. Linear/Nonlinear Non-Gaussian: Using skewed, non-Gaussian noise (Pearson Type III distribution). (3). Simulation demonstration of the bicoherence and time domain Geometric for the Fig.2: after fitting the NNAR model to the real data, only the states close to the equilibrium is be maintained in the model and used to simulate to get spectrum, bicoherence and 2d embedded potential (density) function.*

S.3.1 Simulation for the Hinich's test and BiSCA parametrization demonstration

Here is an example of our bicoherence analysis for different types of signals, we put a simulation demonstration consisting of one channel of real data and 4 corresponding synthetic data (linear Gaussian, linear non-gaussian, nonlinear Gaussian, and nonlinear non-gaussian).

1. Real data: one channel from the MNI atlas data, with Fs=200Hz and 12000 time points samples.
2. Linear Gaussian process: This simulation used an Autoregressive (AR) model with Gaussian innovation.

$$\tilde{x}_{t+1} = \sum_{i=0}^{p-1} \hat{a}_i \tilde{x}_{t-i} + \tilde{u}_t (t = p, \ldots, N+p-1) \tag{1}$$

The AR coefficient is fitted with the real data using Burg's method. In this example, a 45-order AR model is selected with AICc criteria, and the innovation is $N(0,1)$. We simulated $N = 12000$ samples with this AR model, also the same length for the rest 3 simulations.

3. Linear non-gaussian process: Instead of using the Gaussian innovation, here we use the Pearson type III distribution[23] as the innovation enables us to control the first to third-order moment for the distribution. The probability density function is

$$f(u) = \begin{cases} \dfrac{(u-\xi)^{\alpha-1} e^{-(u-\xi)/\beta}}{\beta^{\alpha}\Gamma(\alpha)}, \ \gamma > 0 \\ \dfrac{1}{\sqrt{2\pi}} e^{-\frac{1}{2}\left(\frac{u-\mu}{\sigma}\right)^2}, \ \gamma = 0 \\ \dfrac{(u-\xi)^{\alpha-1} e^{-(\xi-u)/\beta}}{\beta^{\alpha}\Gamma(\alpha)}, \ \gamma < 0 \end{cases} \tag{2}$$

where the parameter of the distribution $\alpha = 4/\gamma^2$, $\beta = \frac{1}{2}\sigma|\gamma|$ and $\xi = \mu - 2\sigma/\gamma$. The parameter μ is the center of distribution, σ is the standard deviation, γ is the skewness. Here we take $\mu = 0$, $\sigma = 1$ and $\gamma = 10$.

4. Nonlinear Gaussian process: Instead of linear p-lag AR model in the linear case, here we used a nonparametric nonlinear autoregressive (NNAR) model to fit the data [24], and simulate 12000 timepoints data recursively. Given timeseries $\{x_t\}_{t=1}^{N_t}$, reconstruct phase space via delay embedding:

$$\boldsymbol{x}_t = [x_t, x_{t-1}, \ldots, x_{t-p+1}]^T \in \mathbb{R}^p \tag{3}$$

The state evolution is modeled as the discrete nonlinear state space model

$$\tilde{\boldsymbol{x}}_{t+1} = F(\tilde{\boldsymbol{x}}_t) + \tilde{\boldsymbol{u}}_t \tag{4}$$

where $F(\cdot):\mathbb{R}^p \to \mathbb{R}^p$ is a p dimensional nonparametric function estimated via adaptive local constant regression (Nadaraya-Waston regression). To simplify the notation to fit regression notation, define embedding vector keys set $\{\boldsymbol{x}_k, \boldsymbol{y}_k\}_{k=1}^{n}$ stored all observed data with $\boldsymbol{x}_k = \boldsymbol{x}_t$ and $\boldsymbol{y}_k = \boldsymbol{x}_{t+1}$, $n = N_t - p - 1$.

$$\hat{F}(\boldsymbol{x}_q^*) = \frac{\sum_{k=1}^{n} K_{h_k}(\boldsymbol{x}_k, \tilde{\boldsymbol{x}}_q)\boldsymbol{y}_k}{\sum_{k=1}^{n} K_{h_k}(\boldsymbol{x}_k, \tilde{\boldsymbol{x}}_q)} \tag{5}$$

$\tilde{\boldsymbol{x}}_q$ is the query vector state during the N-step forward forecasting. The local weights are calculated by the kernel distance $K_{h_k}(\boldsymbol{x}_k, \boldsymbol{x}_q) = \exp\left(-\frac{\|\boldsymbol{x}_k - \boldsymbol{x}_q\|^2}{2h_k^2}\right)$ with bandwidth h_k. The h_k is the hyper parameter of model, in order to handle non-uniform distribution of the states in the p dimension space, we use the κ-th nearest neighborhood $\boldsymbol{x}_{[k,\kappa]}$ of each $\boldsymbol{x}_k$ to determine the local bandwidth

$$h_k = \frac{\|\boldsymbol{x}_{[k,\kappa]} - \boldsymbol{x}_k\|}{\sqrt{p}} \tag{6}$$

To select κ, this paper used a generalized cross-validation (GCV) on the real data $\{x_t\}_{t=1}^{N}$ from case 1, to find the best κ of and record the corresponding h_k for each states $\boldsymbol{x}_k$ in the one-step forward prediction task.

$$\mathrm{GCV}(\kappa) = \frac{\mathrm{MSE}(\kappa)}{(1 - \mathrm{tr}(\boldsymbol{L}(\kappa))/n)^2}$$

where the smoother matrix with elements

$$L_{k,q}(\kappa) = \frac{K_{h_k}(\boldsymbol{x}_k, \boldsymbol{x}_q)}{\sum_{k=1}^{n-1} K_{h_k}(\boldsymbol{x}_k, \boldsymbol{x}_q)}$$

To be computationally efficient, here we use scalar h_k, bandwidth is equal for all dimensions. The embedding dimensions p are used the same as linear simulation case 2 and case 3. The nonparametric function $\hat{F}(\cdot)$ represent all the information of the original (Hankel embedded)

state space or the differential manifold of the system, therefore, we didn't update this nonparametric function during evolution. In the simulation the innovation $\boldsymbol{u}_t^*$ is a multivariable gaussian distribution $\boldsymbol{z}_t$ and scaled with covariance from the residual of the one-step forward prediction $\boldsymbol{u}_t^* = \boldsymbol{L}\boldsymbol{z}_t$, where $\boldsymbol{L}$ is the Cholesky of the covariance matrix of the residual $\boldsymbol{L}\boldsymbol{L}^T = \boldsymbol{\Sigma}$, and $\boldsymbol{\Sigma} = \frac{1}{n-1}\boldsymbol{\epsilon}^T\boldsymbol{\epsilon} + \lambda\boldsymbol{I}_p$ with$\boldsymbol{\epsilon} = \boldsymbol{X} - \hat{\boldsymbol{X}} = [\epsilon_{t,1}, \epsilon_{t,2}, ..., \epsilon_{t,p}]_{t=1}^{n}$.

Nonlinear non-gaussian process: The nonlinear system follows the configuration of case 4. Instead of Gaussian innovation, we use Pearson type III (2) to generate the innovation $\boldsymbol{z}_t$ and then the $\boldsymbol{u}_t^*$.

All 5 cases apply the Multitaper estimation and the BiSCA model fitting with the same parameters as the analysis for real data.

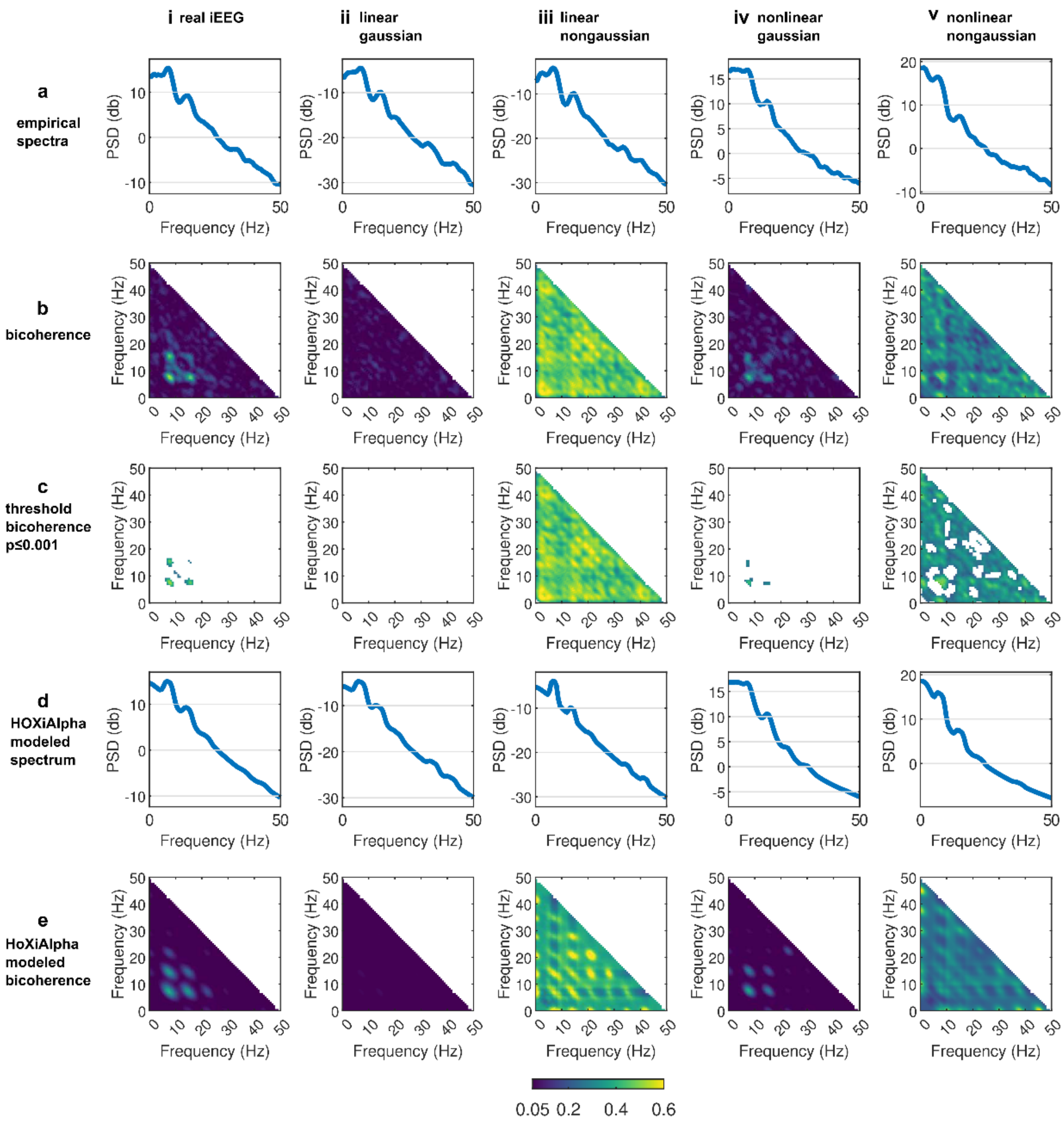

Fig. S 2. Simulation demonstration. *Column from left to right are the 5 cases described in the simulation (i) real iEEG of MNI atlas data; (ii) linear Gaussian; (iii) linear non-Gaussian ; (iv) nonlinear Gaussian; (v) nonlinear non-Gaussian; Row from top to bottom is the result of analysis of each step (a) empirical spectrum calculated with Multitaper method; b) empirical modulus bicoherence calculated with Multitaper method with color scale indicating bicoherence modulus amplitude.; (c) threshold bicoherence with parametric test of p≤0.001: providing a clearer view of which frequency interactions are nonlinearly coupled beyond test level; (d) the spectrum from the fitted BiSCA model; (e) the bicoherence from the fitted BiSCA model.*

Fig. S 2 illustrates how non-Gaussianity and nonlinearity impact neural time series analyses; we synthesized intracranial EEG (iEEG) signals under five distinct scenarios—real iEEG data (MNI atlas), linear Gaussian, linear non-Gaussian, nonlinear Gaussian, and nonlinear non-Gaussian—and compared their power spectrum and bicoherence pattern. The real iEEG data Fig. S 2i exhibits a characteristic 1/f-like power spectrum with modest but significant bicoherence clusters after thresholding ($p \leq 0.001$). This

empirical baseline reveals that genuine neural signals naturally contain nonlinear and Gaussian input features.

In the Fig. S 3 we show the result of statistic test of Fig. S 2 Real iEEG (Top-Left): Both tests are significant, indicating the presence of both nonlinear interactions and non-Gaussian characteristics in the neural data. Linear Gaussian (Middle-Left): As expected for the null case, neither test is significant, correctly identifying the signal as linear and Gaussian. Linear Non-Gaussian (Middle-Right): Only the Gaussian test is significant, demonstrating the method's ability to isolate non-Gaussian properties in the absence of nonlinearity. Nonlinear Gaussian (Bottom-Left): Only the linear test is significant, successfully detecting nonlinear dynamics driven by Gaussian innovations. Nonlinear Non-Gaussian (Bottom-Right): Both tests are significant, correctly identifying the joint presence of nonlinearity and non-Gaussianity. Overall, this figure demonstrates how the combined use of maximum and median bicoherence statistics provides a robust framework for disentangling the distinct contributions of nonlinearity and non-Gaussianity in complex time series.

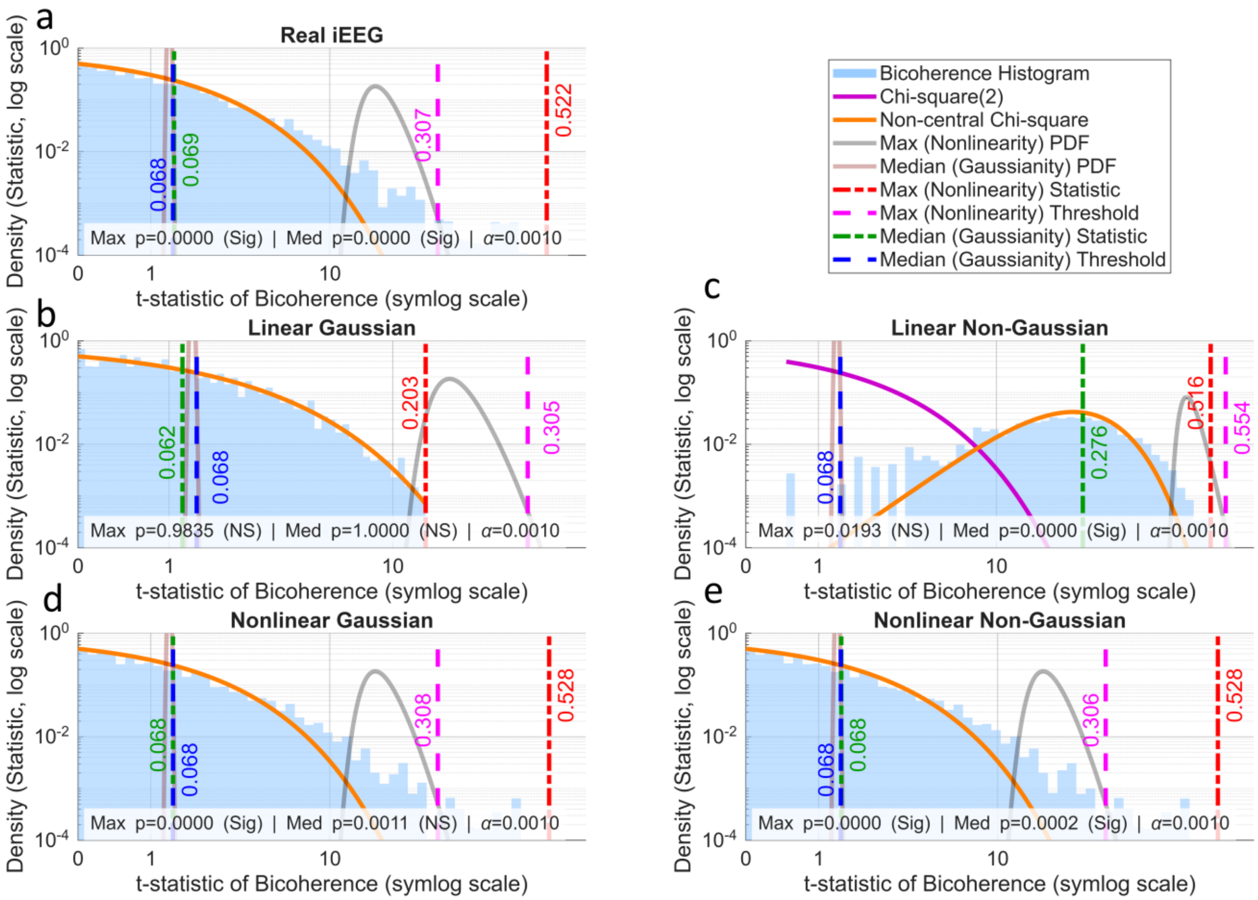

Fig. S 3. Statistical analysis of bicoherence for real and simulated time series. *The figure displays the results of statistical tests applied to the bicoherence of five different time series signals to distinguish between nonlinearity and non-Gaussianity. The five cases are: (Top-left) Real intracranial EEG (iEEG) data; (Middle-left) A simulated linear Gaussian process; (Middle-right) A simulated linear non-Gaussian process; (Bottom-left) A simulated nonlinear Gaussian process; and (Bottom-right) A simulated nonlinear non-Gaussian process. Each subplot shows: Bicoherence Histogram (Light Blue): The distribution of the t-statistic calculated from the empirical bicoherence values of the signal. Chi-square Distributions: The theoretical central chi-square distribution with 2 degrees of freedom (blue line,* $\chi^2(2)$ *), which is expected for a linear Gaussian process. A non-central chi-square distribution (orange line) is fitted to the data, where the non-centrality parameter (*λ_0*) captures deviations from the null hypothesis, related to non-Gaussianity. Statistic PDFs (Gray and Dark Red): The probability density functions for the 'Max' and 'Median' statistics, representing the expected distribution under the null hypothesis. Vertical Lines: The observed values for the 'Max Statistic' (red) and 'Median Statistic' (green) are compared against their respective significance thresholds, 'Max Threshold' (magenta) and 'Median Threshold' (blue). The text at the bottom of each plot summarizes two hypothesis tests: 1. Linear test (through Max): Uses the maximum bicoherence value to test for significant quadratic phase coupling, which indicates nonlinearity. A significant result occurs if the 'Max Statistic' exceeds the 'Max Threshold'. 2. Gaussian test (through median): Uses the median bicoherence value to test for a constant offset in bicoherence, which is characteristic of linear non-Gaussian processes. A significant result occurs if the 'Median Statistic' exceeds the 'Median Threshold'.*

The result shows that the bicoherence can be different even if the second-order spectrum is similar. In the linear non-Gaussian scenario Fig. S 2iii, the data deviate from Gaussianity but do not exhibit intrinsic nonlinear dependencies. This linear yet non-Gaussian structure typically manifests as a uniform or "constant" offset in the bicoherence[25]. In other words, rather than showing localized peaks at specific

bifrequencies, the bicoherence tends to exhibit a more diffuse elevation across the entire frequency domain. Conversely, the bicoherence in the nonlinear Gaussian scenario is limited to well-defined regions (i.e., specific bifrequencies where nonlinearity introduces phase coupling), with otherwise negligible values. This difference underscores that nonlinearity and non-Gaussianity can contribute distinctly to the observed cross-frequency coupling patterns, helping to explain why the bicoherence in the linear non-Gaussian simulation diverges markedly from that of real iEEG data.

We further fitted each case with our BiSCA model, which accurately reproduced both the power spectral density and bicoherence for each scenario (rows of *d* and *e* in Fig. S 2). Notably, the model faithfully recapitulated the real iEEG features, mirroring the 1/f-like spectral decay and the subtle, significant cross-frequency interactions. These results highlight the model's capacity to disentangle and reconstruct complex neural signals, shedding light on the interplay of Gaussianity and nonlinearity that shapes iEEG dynamics.

S.3.2 Simulation for the Geometric view of bicoherence

The simulation of Fig. 2 shows the geometric point of view of the bicoherence. The simulation employed an NNAR method to analyze nonlinear dynamics in intracranial iEEG data containing Wicket wave. The pipeline began with preprocessing a 2-second iEEG segment (sampled at 200 Hz) from a subject with normal variant activity. After reconstructing the phase space using a 23rd-order embedding, the NNAR model was trained to capture the system's dynamics. A pruning step removed non-equilibrium states, retaining only key dynamics with weighted contributions exceeding 1e-2.

$$\mathcal{S} = \left\{ \boldsymbol{x}_k \ \middle|\ \sum_{q=1}^{n_{eq}} w_k^{(q)} > \tau \right\}, \quad w_k^{(q)} = K_{h_k}(\| \boldsymbol{x}_k - \boldsymbol{x}_q^{eq} \|)$$

Deterministic and stochastic forecasts were generated using the pruned model. The deterministic (noise free) simulation to show the 3D phase portrait skeleton visualization, spectrum and bicoherence of this limit cycle. The stochastic forecasts with innovations modeled as Gaussian noise scaled by residual covariance to obtain potential landscape of 2D state space. The system's geometry was projected into 2D/3D state spaces via PCA of the high dimensional states set $\mathcal{S}$, and potential landscapes were estimated using kernel density methods on 100×100 grids, this is a nonparametric version of the landscape estimation[26]. Spectrum and bicoherence, was implemented also with Multitaper estimators ($NW = 2.5$) across 300-sample windows with 75% overlap.

To contrast with the asymmetric dynamics, we simulated symmetric nonlinear oscillations by enforcing the nonparametric state evolution function to satisfy $F(-\boldsymbol{x}) = -F(\boldsymbol{x})$, making the system dynamics invariant under state inversion $(\mathbf{X} \to -\mathbf{X})$. The modified state evolution becomes:

$$\tilde{\boldsymbol{x}}_{t+1} = \frac{F(\tilde{\boldsymbol{x}}_t) - F(-\tilde{\boldsymbol{x}}_t)}{2} + \tilde{\boldsymbol{u}}_t$$

where the averaging ensures cancellation of even-order nonlinearities.

The reconstructed phase portrait revealed a stable limit cycle (Fig. 2C), confirming the system's oscillatory nature. The potential landscape exhibited asymmetric minima (Fig. 2I), explaining the spikes' directional morphology. Spectral analysis showed harmonically related peaks at 8.7Hz and 17.4Hz (Fig. 2J), with total harmonic distortion (THD) -4.08dB. Bicoherence analysis demonstrated significant phase coupling between fundamental and harmonic components (Fig. 2K), quantifying the nonlinear interactions. The symmetric version of the system, depicted in subplot (B) to (F), provides a contrasting perspective to the asymmetric case. Besides the symmetricity of the time domain analysis (B) to (D), the spectrum shows it only has the odd order harmonics, therefore, the bicoherence shows zero everywhere.

The asymmetric bicoherence profile (Fig. 2K) reflects directionally biased nonlinear interactions, characteristic of depolarization-hyperpolarization asymmetry in the neuronal networks. The reconstructed limit cycle (Fig. 2H) suggests possible self-sustaining oscillations stabilized by voltage-gated ion channel dynamics during the sample time interval. The potential landscape's asymmetry (Fig. 2) aligns with the greater energy required for spike initiation versus termination, consistent with sodium channel inactivation kinetics. The 9 Hz spectral peak corresponds to thalamocortical resonance frequencies implicated in spike-wave generation. While the model successfully captured short-term dynamics ($\tau < 100$ ms), longer simulations showed phase drift, this could be introduced by the step of pruning removed the activities shifted by the slow variables, the underlying attractor may not perfect limit cycle but quasiperiodic or chaotic, suggesting the need for additional slow variables in future extensions [27,28]. These analysis bridges the spectral domain nonlinear analysis with the time domain evolution, providing a framework for quantifying stability landscapes in the nonlinear oscillations

The geometric interpretation of bicoherence provides critical insights into system dynamics. For an asymmetric system (Fig. 2G-K), significant bicoherence between the fundamental frequency (8.7 Hz) and its second harmonic (17.4 Hz) signifies quadratic phase coupling. This interlocking of phases constrains

the system's trajectory in phase space to a specific manifold, manifesting as an asymmetric "wicket" waveform and a distorted limit cycle in the phase portrait. Conversely, for a symmetric system (Fig. 2B-F), the bicoherence is zero, reflecting the absence of phase coupling. The system's trajectory is unconstrained, resulting in a uniform limit cycle and a time-domain waveform with only odd-order harmonics. This contrast highlights how bicoherence geometrically quantifies the degree of asymmetry in the system's underlying potential landscape. Furthermore, bicoherence helps distinguish system-based nonlinearity from non-Gaussian noise. In a nonlinear system, the energy landscape imposes state-dependent constraints, suppressing noise-induced perturbations more effectively in regions with strong recovery forces (e.g., steep potential wells). This state-specific "filtering" of non-Gaussian inputs reduces the measured bicoherence value (b(L)=0.0161) compared to a linear system, where such perturbations propagate homogeneously (Fig. 2). This distinction underscores the importance of modeling both system nonlinearity and noise non-Gaussianity when interpreting higher-order spectral features.

The geometric interpretation of bicoherence reveals critical insights into the nonlinear dynamics of the system, particularly in how phase coupling shapes its oscillatory behavior. In the asymmetric system, the bicoherence plot (Fig. 2K) shows significant phase coupling between the fundamental frequency at 8.7 Hz and its second harmonic at 17.4 Hz, indicating quadratic nonlinear interactions that constrain the system's trajectory in phase space to a manifold reflecting these phase-locked oscillations. This interlocking of phases manifests as the asymmetric morphology in the time-domain waveform of the Wicket (Fig. 2A), with the directional bias corresponding to imbalanced phase relationships. The reconstructed phase portrait (Fig. 2H) supports this, as these nonlinear constraints shape the stable limit cycle's trajectory. Conversely, in the symmetric system, the bicoherence is zero everywhere (Fig. 2F), aligning with the regular, balanced oscillations in the time domain (Fig. 2B) and the presence of only odd-order harmonics (Fig. 2E). Here, the absence of phase coupling results in an unconstrained trajectory in phase space, producing a more uniform limit cycle (Fig. 2C). This contrast highlights how bicoherence geometrically quantifies the degree of nonlinearity and asymmetry in the system's dynamics, linking spectral domain insights to the time-domain evolution and the underlying potential landscape (Fig. 2D and Fig. 2I). These findings underscore the utility of bicoherence in analyzing stability and morphology in nonlinear oscillatory systems, offering a framework to explore the interplay between structure and dynamics.

S.3.3 Forward-Model Simulation and Mechanistic Interpretation of Elevated Sensor-Level Nonlinearity

The higher prevalence of significant nonlinearity in scalp EEG (81.6%) compared to iEEG (67.9%) requires mechanistic explanation. Two factors are relevant. First, non-sinusoidal alpha and mu waveforms are a direct manifestation of quadratic nonlinearity: peak-trough asymmetry generates even harmonics and corresponding bicoherence signatures [29,30]. Mu rhythms exhibit significantly stronger peak-trough asymmetry than alpha rhythms [31], and non-sinusoidal alpha can generate narrow-band harmonic peaks in bicoherence [32]. Because these nonlinear sources are spatially focal (primarily parietal and occipital cortex), the second factor — volume conduction — determines how many sensor channels detect them. Volume conduction in scalp EEG spreads focal nonlinear activity across many channels, increasing the fraction that exceed significance thresholds. Scalp-level bicoherence is strongly affected by this spatial mixing, with substantial differences between source and sensor estimates [33] for instance, frontal sensors can reflect up to 75% contribution from occipital and central sources [34]. Univariate normalization can mitigate coupling-dependent artifacts in bicoherence estimation [35].

Both factors predict regional variation in nonlinearity prevalence: bicoherence should be strongest in posterior regions where alpha and mu rhythms dominate, and weakest in frontal cortex where alpha power is low. To examine this prediction in the empirical data, we pooled all contacts (iEEG) or channel-subject pairs (EEG) by brain region and computed GL/NGL/GNL/NGNL classification proportions (Fig. S 4). For iEEG, the 39 fine-grained anatomical regions from the MNI atlas are merged into 8 lobe-level groups (Frontal, Temporal, Parietal, Occipital, Insular, Cingulate, Limbic/MTL, Central Operculum). For EEG, the 19 standard 10-20 channels are grouped into 5 scalp regions (Frontal: 7 channels; Central: 3; Temporal: 4; Parietal: 2; Occipital: 2).

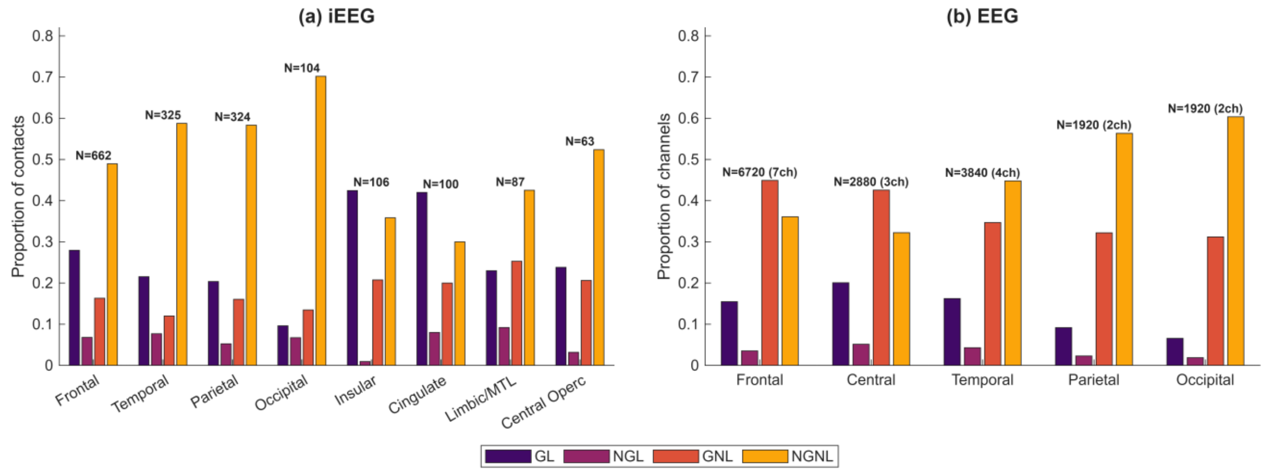

Fig. S 4. Regional proportions of GL, NGL, GNL and NGNL classifications in iEEG and scalp EEG. *a, iEEG contacts pooled across all subjects and grouped into frontal, temporal, parietal, occipital, insular, cingulate, limbic/medial temporal lobe (MTL) and central opercular regions. b, scalp EEG channel-subject pairs pooled across all subjects and grouped into frontal, central, temporal, parietal and occipital regions. Bars show the proportion of recordings in each class within each region, and numbers above groups indicate the number of contacts or channel-subject pairs (with the number of contributing EEG channels in parentheses). NGNL predominates across most iEEG regions, whereas EEG shows relatively greater GNL in anterior regions and greater NGNL in parietal and occipital regions. GL, Gaussian linear; NGL, non-Gaussian linear; GNL, Gaussian nonlinear; NGNL, non-Gaussian nonlinear.*

Sources are defined on a 2003-vertex cortical mesh (Desikan-Killiany atlas, 2k resolution). For each vertex $j = 1, \dots, N_{\text{src}}$ ($N_{\text{src}} = 2003$), a background aperiodic process is generated as an AR(1) process:

$$E_j(t) \sim \mathcal{N}(0, \sigma_{\text{bg}}^2), \quad S_j^{\text{bg}}(t) = a\, S_j^{\text{bg}}(t-1) + E_j(t)$$

where $a = 0.98$ is the AR coefficient and $\sigma_{\text{bg}} = 1.0$ the driving noise amplitude. Each background source is then standardized to zero mean and unit variance. All simulations used a fixed random seed (rng(1)) for exact reproducibility.

Two types of oscillatory sources are injected into specific cortical regions of interest (ROIs):

A quadratic nonlinearity generates waveform asymmetry and harmonic content placed on parietal as Nonlinear mu source:

$$S_\mu(t) = \sin(2\pi f_\mu t) + \kappa \cdot [\sin(2\pi f_\mu t)]^2 + \sigma_\mu\, \eta_\mu(t)$$

where $f_\mu = 11$ Hz, $\kappa = 0.6$ controls the quadratic coupling strength, $\sigma_\mu = 0.5$ is the source noise amplitude, and $\eta_\mu(t) \sim \mathcal{N}(0,1)$. The signal is standardized to zero mean and unit variance after generation.

No quadratic nonlinearity placed on occipital and frontal Single-tone alpha source:

$$S_\alpha(t) = \sin(2\pi f_\alpha t) + \sigma_\alpha \, \eta_\alpha(t)$$

where $f_\alpha = 10$ Hz and $\sigma_\alpha = 0.05$. The signal is likewise standardized.

We note that modeling the alpha source as a single-tone sinusoid does not imply alpha oscillations are linear in general—symmetric (odd-order) nonlinear systems (e.g., Stuart-Landau limit cycles with cubic dynamics) can generate near-sinusoidal waveforms with negligible bicoherence. The simulation isolates the effect of quadratic nonlinearity on bicoherence detection.

The injected sources are additively superimposed on the background at ROI vertices defined by the Desikan-Killiany atlas:

ROI	Atlas Labels	Injected Signal	Vertices
Parietal (mu)	Inferior parietal L/R, Superior parietal L/R, Postcentral L/R	$S_\mu(t)$ (nonlinear)	~300
Occipital (alpha)	Pericalcarine L/R, Cuneus L/R, Lingual L/R	$S_\alpha(t)$ (single-tone)	~200
Frontal (alpha)	Superior frontal L/R	$S_\alpha(t)$ (single-tone)	~150

For each ROI vertex j:

$$S_j(t) = S_j^{\text{bg}}(t) + S_{\text{injected}}(t)$$

Vertices outside all ROIs retain only the background AR(1) process.

Sensor-level signals are computed as linear mixtures of cortical sources via realistic leadfield matrices, without additional sensor noise:

$$\mathbf{X}_{\text{EEG}}(t) = L_{\text{EEG}}\,\mathbf{S}(t), \quad \mathbf{X}_{\text{iEEG}}(t) = L_{\text{iEEG}}\,\mathbf{S}(t)$$

$L_{\text{EEG}} \in \mathbb{R}^{19\times 2003}$: OpenMEEG BEM headmodel on the same 2k cortical grid. The original gain matrix $G \in \mathbb{R}^{N_{\text{ch}}\times 3N_{\text{src}}}$ (three orientations per source) is collapsed to a scalar leadfield along the cortical surface normal:

$$L_{\text{EEG}}(i,j) = G_j^{(i)} \cdot \hat{n}_j$$

where $G_j^{(i)} \in \mathbb{R}^{1\times 3}$ is the gain for channel i at source j and $\hat{n}_j$ is the surface-normal orientation vector from GridOrient.

$L_{\text{iEEG}} \in \mathbb{R}^{1766\times 2003}$: Super-subject leadfield constructed by vertically stacking per-subject leadfield matrices from all 106 subjects in the Frauscher et al.(2018)[1] atlas. Channels with all-zero leadfield rows (no sensitivity to any cortical source) are excluded, yielding 1766 channels. The empirical analysis uses a different preprocessing pipeline and retains 1,771 channels.

Both leadfield matrices use raw amplitudes (no row-normalization). The observed signal at each channel is the linear combination of all cortical source signals weighted by the leadfield row.

The simulation parameters are summarized below.

Parameter	Symbol	Value
Sampling rate	F_s	200 Hz
Signal length	N	11,600 samples (58 s)
Random seed	—	1 (deterministic)
AR(1) coefficient	a	0.98
Background noise amplitude	σ_{bg}	1.0
Mu fundamental frequency	f_μ	11 Hz
Alpha fundamental frequency	f_α	10 Hz
Quadratic coupling strength	κ	0.6
Mu source noise	σ_μ	0.5
Alpha source noise	σ_α	0.05
Source grid vertices	N_{src}	2003
EEG channels	$N_{\text{ch}}^{\text{EEG}}$	19
iEEG channels (super-subject)	$N_{\text{ch}}^{\text{iEEG}}$	1766

Per channel, bispectrum was estimated with multitaper settings matching the main analysis of manuscript. Key result (max-statistic significance): iEEG 30.9% vs EEG 94.7% significant channels. This indicates that, even with identical underlying sources, scalp-level spatial mixing substantially increases detectability of quadratic phase coupling.

The simulation channel counts and empirical channel counts differ slightly due to modality-specific leadfield construction and exclusion criteria; however, significance is assessed independently per channel, so interpretation is based on per-channel detection rates rather than absolute channel totals. The resulting channel-wise distributions of the nonlinearity and Gaussianity statistics are shown in **Fig. S 5**.

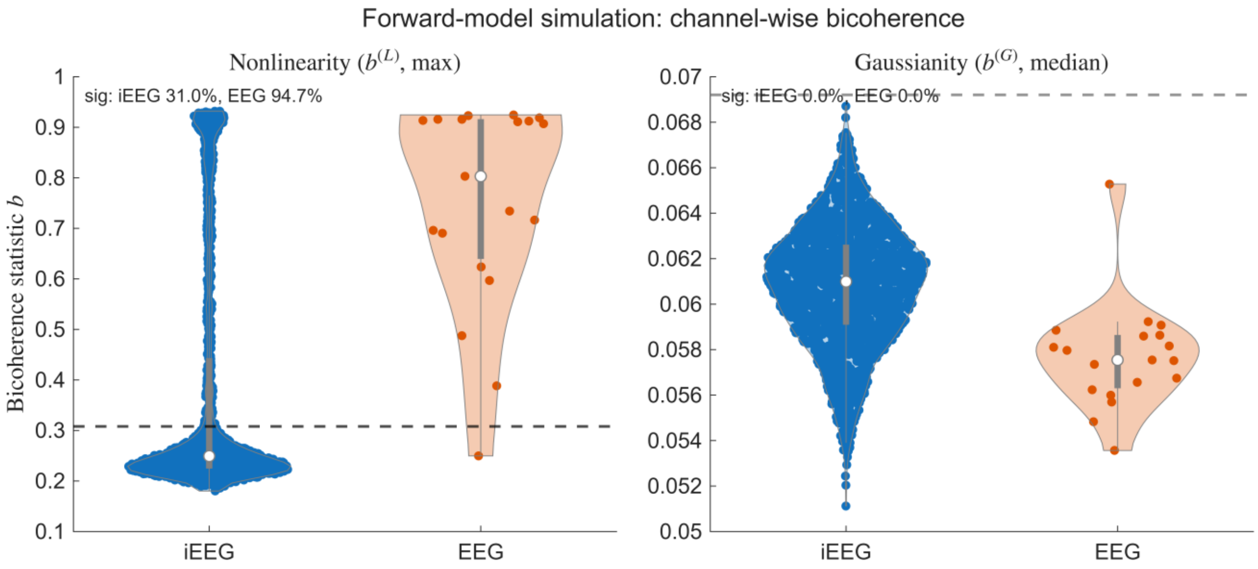

Fig. S 5. Violin plot of channel-wise bicoherence statistics for iEEG and EEG. *Left pair: nonlinearity ($b^{(L)}$, max statistic); right pair: Gaussianity ($b^{(G)}$, median statistic). Dashed lines mark significance thresholds ($\alpha = 0.001$). EEG shows 94.7% channels significant for nonlinearity vs. 30.9% in iEEG; neither modality shows significant non-Gaussianity (0.0%).*

Even with identical underlying cortical sources, spatial mixing inherent to scalp EEG produces a substantially larger proportion of channels labeled as nonlinear (94.7% vs. 30.9%). The channel count asymmetry (19 EEG vs. 1,766 iEEG) mirrors the inherent difference between these recording modalities and is consistent with the empirical data (19 scalp EEG channels vs. 1,771 iEEG channels); importantly, each channel's bicoherence significance is tested independently, so the per-channel test is unaffected by the total number of channels. This confirms that the higher prevalence of significant nonlinearity in EEG compared to iEEG (81.6% vs. 67.9% in the empirical data) is consistent with volume conduction amplifying the detectability of quadratic phase coupling at the sensor level.

S.4 Model Adequacy, Goodness of Fit, and Parameter Accounting

Section S.4 gathers the practical evidence that the BiSCA parametrization is numerically adequate for the empirical data. The section proceeds from observed fit quality to the parameter bookkeeping needed to interpret AIC-based model selection.

S.4.1 Goodness of fit

To demonstrate how much variance can be explained by the model, here we put the cumulative density function of the R^2. In the formula defined in formula (13) and (15), the parameter $\{\mu_{\rho,k}\}$ has two

options: 1) Harmonic fixed with the harmonic relationship of the "fundamental oscillation", 2) Free: initialize with spectrum and optimize the joint fit. We put the R^2 of both cases here.

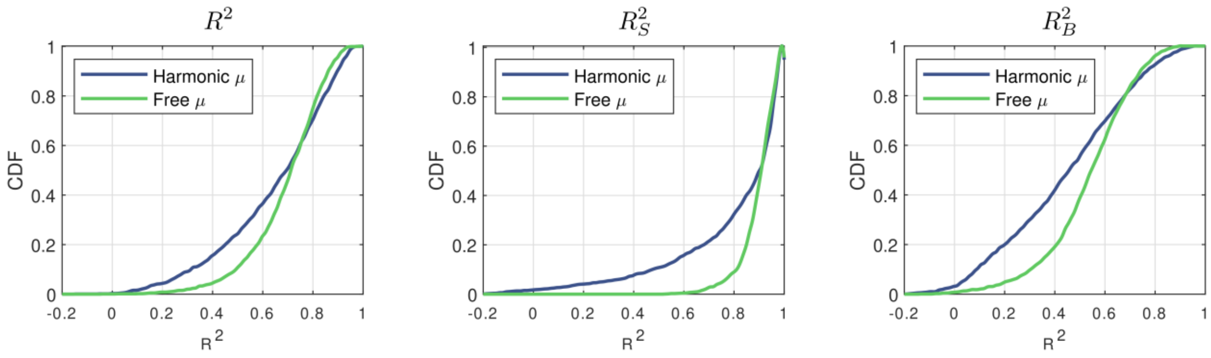

Fig. S 6 Distributions of the coefficient of determination R^2 ***in three different conditions.*** *a) is the* R^2 *of the full model; b) is the* R^2 *for the spectrum; c) is the* R^2 *for the bispectrum*

The R^2 distribution is calculated for the fit of each channel shown in the Fig. S 6. The goodness of fit suggests that the model's goodness of fit varies considerably across the entire dataset. The "free" option of the peak center fits with a greater degree of freedom. Therefore, its R^2 is concentrated on the right side, compared to the "harmonic" organized center peak option. Some phenomena, such as split Alpha, may not be described by the "harmonic" organized model.

Fig. S 6A shows a broad distribution of overall R^2 values, suggesting that the model's goodness of fit varies considerably across the entire dataset. A noticeable upward trend toward higher R^2 (up to ≈ 1) indicates that many data segments exhibit strong fits, although a small portion remains at the lower end of the scale. Since the variance of the spectrum and the variance of the bispectrum is quite different, which also vary with subject the R_S^2 and R_B^2 are more intuitive. Fig. S 6B R_S^2 are comparatively narrower near the high value but rise sharply toward $R_S^2 \approx 1$. This pattern implies that many single-subject spectral fits are quite robust, with fewer cases in the middle range of goodness of fit. Fig. S 6C presents a more bell-shaped distribution of baseline R_B^2 values. Most of these are clustered around moderate goodness-of-fit levels $R_B^2 \approx 0.4 - 0.6$, with fewer instances at the extremes (near 0 or near 1). Overall, bispectrum fits appear to be consistent but rarely reach near-perfect levels.

S.4.2 Model-order and parameter accounting

Our BiSCA Model selection uses AIC to choose the number of oscillatory components. To make the notation explicit, it is necessary to distinguish the oscillatory component count, K, from the total free-parameter count, N_p, that enters the AIC penalty term $2k - 2\ln L$.

Across $N = 1{,}771$ iEEG channels, the AIC-selected models show a median of five fitted peaks above 5% spectral power, with an interquartile range of 3 to 6. A representative worked example with $k_s = 0$, $k_h = 3$, and $K = 3$ gives an internal peak count $K_{\text{int}} = 4$, bispectral grid dimension $N_1 = 5$, spectrum parameter count $N_{p,S} = 14$, bispectrum parameter count $N_{p,B} = 20$, and total free-parameter count $N_p = 34$.

For the frequency grid used in the analysis, $N_f = 61$, the effective sample-size term is

$$N_e = N_f + (N_f^2 + N_f)/2 = 1952,$$

so $N_e/N_p \approx 57$. This ratio indicates that the AIC penalty is applied in a regime where the effective sample size remains much larger than the number of fitted parameters.

S.5 Peak relation from the spectrum

Section S.5 links two related questions: how peak relations should be interpreted empirically, and how admissible peak interactions are formalized in the BiSCA bispectral model.

S.5.1 Peak relation from the spectrum

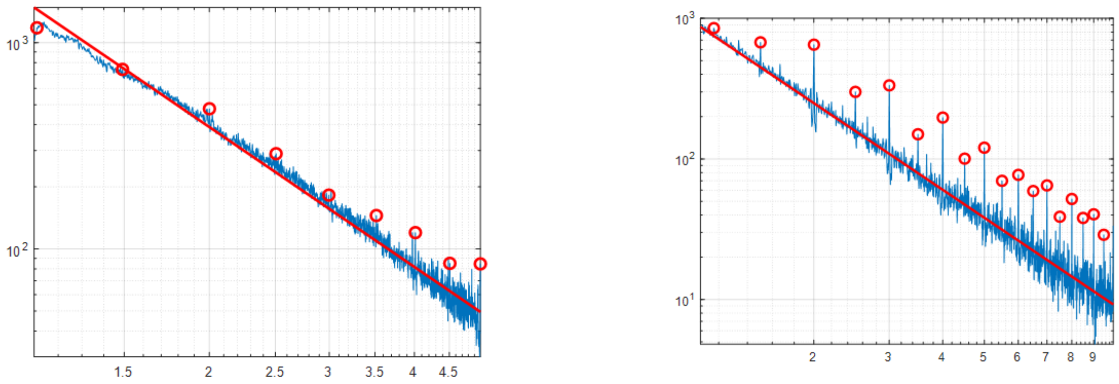

Fig. S 7. the ratio of the peaks identified in the spectrum. *The left is from harmonized 1563 × 19 channels scalp EEG data. The right is from 1771 channels of iEEG data.*

The empirical distributions of the peak ratios identified in scalp EEG and iEEG are shown in Fig. S7. Beyond integer harmonic peaks of periodic oscillations, other proportions between peak frequency reveal possible quasi-periodic motion and chaotic; the distribution of the proportions showed possible scale free of the organization of peaks. On the Exponent Scale, the slope is 2.2531 for EEG and 2.0493 for iEEG. However, metaphysical models attempt to impose intriguing physical interpretations on empirical data without necessarily ensuring that these interpretations adhere to rigorous statistical or biophysical validity. Evertz et al. (2022)[36] point out correctly that there is doubt that the aperiodic component is either "scale-free" or a reflection of criticality. An alternative is to look at biophysical models with effects of the extracellular space [37] as mentioned by Evertz et al. (2022)[36]. In addition to interest and questions about the scale-free nature of Xi process [38]. This question was discussed in Valdés-Sosa et al. in 1999[24,39], when people argue that the EEG is chaos, the neural activity is a nonlinear stochastic system driven by the noise. We reserve these possibilities, and these conclusions still need to be verified by rigorous statistical methods. One possibility is that using the bicoherence in this paper to study the relationship between the components is possible when all the ratios here are the intermodulation combination of the integer peaks.

Beyond integer harmonic peaks, the empirical spectrum can exhibit additional frequency ratios suggestive of quasi-periodic structure, intermodulation relationships, or broader scale-dependent organization. This observation motivates a cautious interpretation of peak relations: some ratios may reflect meaningful coupling structure, whereas others may reflect the joint organization of oscillatory and aperiodic components without implying a single harmonic mechanism.

S.5.2 Closure-constrained BiSCA parametrization

For that reason, the present work does not force every observed peak into a universal harmonic interpretation. Instead, explicit closure constraints are introduced only when the bispectrum provides evidence for admissible interactions.

BiSCA models the oscillatory bispectrum on the harmonic closure manifold using a pair-indexed parametrization,

$$\hat{B}_\rho(f_1, f_2) = \sum_{(m,n)\in\mathcal{P}} h_{B,m,n}\ t_2(f_1, f_2;\ \mu_m, \mu_n) \qquad \text{S.1}$$

where $\mathcal{P}$ is the set of closure-admissible peak pairs and $h_{B,m,n} \in \mathbb{C}$. The product kernel follows from the Volterra bispectrum structure[40,25,41]:

$$t_2(f_1, f_2;\, \mu_m, \mu_n) = t(f_1;\, \mu_m)\, t(f_2;\, \mu_n)\, t(f_1 + f_2;\, \mu_m + \mu_n)$$

The bispectrum lives on the closure manifold $\{(f_1, f_2, f_1 + f_2)\}$, so only triads satisfying $\mu_m \approx \mu_k + \mu_l$ produce non-negligible contributions. Under the harmonic constraint $\mu_k = k\, f_0$, the closure condition reduces to integer arithmetic k + l = m. The remainder of this section derives the reduction from the general triple-indexed expansion to Eq. S.1, enumerates the admissible terms for specific values of K, and provides an empirical verification in 3D frequency space.

Bispectral closure. The bispectrum of a univariate stationary time series is a function of two frequencies, not three. For a zero-mean stationary process, the third-order cumulant depends on two lags:

$$c_3(\tau_1, \tau_2) = \mathrm{Cum}[x(t),\, x(t+\tau_1),\, x(t+\tau_2)] = \mathbb{E}[x(t)\, x(t+\tau_1)\, x(t+\tau_2)]$$

Its two-dimensional Fourier transform defines the bispectrum (Nikias & Petropulu, 1993, Eq. 2.30; Brillinger, 1965)[25,42]:

$$C_3^x(\omega_1, \omega_2) = \sum_{\tau_1=-\infty}^{\infty} \sum_{\tau_2=-\infty}^{\infty} c_3\,(\tau_1, \tau_2)\, e^{-j(\omega_1\tau_1 + \omega_2\tau_2)}$$

In the frequency domain, stationarity enforces a Kronecker/Dirac delta constraint on the triple product of Fourier coefficients:

$$\mathbb{E}[X(\omega_1)\, X(\omega_2)\, X^*(\omega_3)] = C_3^x(\omega_1, \omega_2) \cdot \delta(\omega_1 + \omega_2 - \omega_3)$$

The delta $\delta(\omega_1 + \omega_2 - \omega_3)$ forces $\omega_3 = \omega_1 + \omega_2$. To see why, substitute the spectral representation $x(t) = \Sigma\, X(\omega) \exp(j\omega t)$ into the third moment $E[x(t)\, x(t+\tau_1)\, x(t+\tau_2)]$ and sum over t: the time summation produces $\delta(\omega_1 + \omega_2 - \omega_3)$. In Brillinger & Rosenblatt's (1967b)[43] spectral representation (their Eq. 2.14, p. 192), this delta appears explicitly in the definition of the k-th order spectral density, establishing $\Sigma\, \lambda_j \equiv 0$ as the mathematical expression of the closure manifold. Writing $B(f_1, f_2) = E[X(f_1)\, X(f_2)\, X^*(f_1+f_2)]$ is not an approximation—it is the definition, with $f_3 = f_1 + f_2$ enforced by stationarity.

In Cosmic Microwave Background or turbulence studies, three-point correlations involve three wavevectors in 2D or 3D space, and the closure condition reduces dimensionality but does not collapse to a simple pair index. For univariate time series, each frequency is a scalar, so $f_3 = f_1 + f_2$ fully determines the third quantity from the first two (Komatsu & Spergel, 2001)[44].

Triple sum and closure condition. Under the Xi and Rho independence assumption (Section S.6.3), the bispectrum decomposes as $B_x = B_\xi + B_\rho$. The oscillatory signal $x_\rho(t) = \Sigma_m x_{\rho,m}(t)$ consists of K narrowband components centered at frequencies μ_m. Expanding in the bispectrum definition:

$$B_\rho(f_1, f_2) = \sum_{m=1}^{K}\sum_{n=1}^{K}\sum_{k=1}^{K} \mathbb{E}\left[X_{\rho,m}(f_1)\, X_{\rho,n}(f_2)\, X_{\rho,k}^{*}(f_1 + f_2)\right]$$

This is the general triple-index expansion: K^3 terms in general. By construction (Eq. 13 in the manuscript), each component $x_{\rho,m}$ has spectral energy concentrated near μ_m within a bandwidth of order $\sim 2\sigma_\rho$. For a given (f_1, f_2), the (m, n, k)-th term involves the product:

$$\underbrace{t\,(f_1; \mu_m)}_{large\ \ only\ \ if\ \ f_1 \approx \mu_m} \quad \cdot \quad \underbrace{t\,(f_2; \mu_n)}_{large\ \ only\ \ if\ \ f_2 \approx \mu_n} \quad \cdot \quad \underbrace{t\,(f_1 + f_2; \mu_k)}_{large\ \ only\ \ if\ \ f_1 + f_2 \approx \mu_k}$$

All three factors must be simultaneously non-negligible. The first two factors constrain (f_1, f_2) to the neighborhood of (μ_m, μ_n). Given this, $f_1 + f_2 \approx \mu_m + \mu_n$. The third factor then requires:

$$\mu_k \approx \mu_m + \mu_n$$

Once (m, n) is chosen, k is forced to be whichever component has center frequency closest to $\mu_m + \mu_n$. If no such component exists, the third factor evaluates to approximately zero and the entire term is negligible. The triple sum can therefore be rewritten by replacing the free index k with the constrained value k*(m, n):

$$B_\rho(f_1, f_2) \approx \sum_{m=1}^{K}\sum_{n=1}^{K} \mathbb{E}\left[X_{\rho,m}(f_1)\, X_{\rho,n}(f_2)\, X_{\rho,k^*}^{*}(f_1 + f_2)\right] \cdot 1[\mu_m + \mu_n \approx \mu_{k^*}]$$

The inner sum over k has collapsed: only one value of k contributes for each (m, n). No algebraic symmetry of the tensor $E[X_m X_n X_k^*]$ reduces 3 independent indices to 2. The reduction is a physical consequence: the narrowband kernel product acts as a selector that forces k ≈ m + n, leaving only 2 free indices. Terms with k ≠ m + n are not zero by symmetry—they are negligible because the kernel product vanishes.

Harmonic constraint. Under the harmonic peak model $\mu_m = m\, f_0$, the approximate condition $\mu_k \approx \mu_m + \mu_n$ becomes exact:

$$m\,f_0 + n\,f_0 = k\,f_0 \quad \Leftrightarrow \quad m + n = k$$

An admissible triad is any (m, n, k) with m, n ∈ {1, …, K} and k = m + n ≤ K. The LTI transfer-function structure confirms this: Nikias & Petropulu (1993, Eq. 2.80)[25] show that for a linear system with white noise input,

$$C_3^y(\omega_1, \omega_2) = \gamma_3^x \cdot H(\omega_1) \cdot H(\omega_2) \cdot H^*(\omega_1 + \omega_2)$$

where the transfer function is evaluated at two free frequencies and their sum. The sum frequency $\omega_1 + \omega_2$ is not a free variable—it is determined. The component-index reduction is the discrete, narrowband specialization of this same principle.

Table S 7. Continuous vs. discrete bispectral closure.

	**Continuous (Brillinger; Nikias)**	**Discrete (BiSCA)**
Signal	Continuous spectrum	K narrowband peaks
Constraint	$\omega_3 = \omega_1 + \omega_2$	$\mu_k = \mu_m + \mu_n$
Free variables	(ω_1, ω_2); ω_3 determined	(m, n); k determined
Reduction	3D integral → 2D	triple sum → double sum

For typical BiSCA parameters ($\sigma_p / f_0 \approx 0.1$, Lorentzian kernel), a non-admissible triad whose sum frequency $\mu_m + \mu_n$ misses the nearest peak by $\Delta f = f_0$ experiences a kernel suppression factor of $t(f_0; 0, \sigma_p) = [1 + (f_0/\sigma_p)^2]^{-1} \approx 10^{-2}$. In the non-admissible triple product, only the third factor is off-center; the first two remain well-centered. The product kernel for non-admissible triads is thus suppressed by roughly two orders of magnitude. For the EEG data analyzed in this study, $\sigma_p / f_0 \approx 0.05$–$0.15$, placing the analysis within the narrowband regime.

Worked example: K = 2. Peaks at $\mu_1 = f_0$ and $\mu_2 = 2f_0$. The triple sum has $K^3 = 8$ terms. The closure condition—not a symmetry argument—eliminates 7 of the 8:

Table S 8. All 8 triple-sum terms for K = 2.

**m**	**n**	**k**	**m+n vs k**	**Status**

1	1	1	2 ≠ 1	negligible
**1**	**1**	**2**	**2 = 2**	**admissible**
1	2	1	3 ≠ 1	negligible
1	2	2	3 ≠ 2	negligible
2	1	1	3 ≠ 1	negligible
2	1	2	3 ≠ 2	negligible
2	2	1	4 ≠ 1	negligible
2	2	2	4 ≠ 2	negligible

Of the 8 terms, exactly 1 satisfies the closure: (m, n, k) = (1, 1, 2), corresponding to the single pair (m, n) = (1, 1). The formal double sum in Eq. S.1 runs over m = 1, …, K and n = 1, …, m, giving K(K+1)/2 = 3 formal pairs:

Table S 9. All formal pairs in Eq. S.1 for K = 2 and their closure status.

**Pair (m, n)**	**Sum freq μ_m+μ_n**	**Peak at sum freq?**	**Status**
(1, 1)	$2f_o = \mu_2$	Yes (peak at μ_2)	admissible
(2, 1)	$3f_o$	No (exceeds $\mu_k = 2f_o$)	kernel ≈ 0
(2, 2)	$4f_o$	No	kernel ≈ 0

Eq. S.1 formally sums over 3 pairs, but the product kernel is negligible for pairs (2, 1) and (2, 2) because the third factor finds no spectral peak to localize around. Thus, for K = 2: 8 triple-sum terms → 1 closure-admissible triad → 1 active pair in the double sum (out of 3 formal pairs).

Worked example: K = 6. Peaks at $\mu_m = m\ f_o$ for m = 1, …, 6. The triple sum has K^3 = 216 terms. An admissible triad requires m + n = k with 1 ≤ m, n ≤ 6 and k ≤ 6:

Table S 10. All admissible triads for K = 6, grouped by sum index k = m + n.

**k**	**Triads (m, n, k)**	**Count**
2	(1,1,2)	1
3	(1,2,3), (2,1,3)	2
4	(1,3,4), (3,1,4), (2,2,4)	3
5	(1,4,5), (4,1,5), (2,3,5), (3,2,5)	4
6	(1,5,6), (5,1,6), (2,4,6), (4,2,6), (3,3,6)	5
	**Total admissible triads**	**15**

Since k = m + n is determined by (m, n), each triad maps to a unique pair. Pairs (m, n) and (n, m) contribute to the same bifrequency region by the permutation symmetry $B(f_1, f_2) = B(f_2, f_1)$. Restricting to n ≤ m:

Table S 11. Unique closure-admissible pairs for K = 6.

**Pair (m,n)**	**n ≤ m**	**Sum k=m+n**	**Triads merged**	**Bifreq. center**
(1,1)	✓	2	(1,1,2)	(f_o, f_o)
(2,1)	✓	3	(1,2,3)+(2,1,3)	$(2f_o, f_o)$
(3,1)	✓	4	(1,3,4)+(3,1,4)	$(3f_o, f_o)$
(4,1)	✓	5	(1,4,5)+(4,1,5)	$(4f_o, f_o)$
(5,1)	✓	6	(1,5,6)+(5,1,6)	$(5f_o, f_o)$
(2,2)	✓	4	(2,2,4)	$(2f_o, 2f_o)$
(3,2)	✓	5	(2,3,5)+(3,2,5)	$(3f_o, 2f_o)$
(4,2)	✓	6	(2,4,6)+(4,2,6)	$(4f_o, 2f_o)$
(3,3)	✓	6	(3,3,6)	$(3f_o, 3f_o)$

**Total unique pairs**	**9**

The three counts—216, 15, 9—describe successive stages of the reduction: 216 → 15 (closure), 15 → 9 (permutation symmetry). The formal pair count in Eq. S.1 is K(K+1)/2 = 21, of which 9 are closure-admissible and 12 are kernel-suppressed (m + n > K).

Table S 12. Parameter count comparison for K = 6.

**Quantity**	**Count**	**Explanation**
Triple-sum terms (K^3)	216	General expansion
Closure-admissible triads	15	m + n = k ≤ K, all orderings
Unique pairs (n ≤ m)	9	After permutation symmetry
Formal pairs in Eq. S.1	21	K(K+1)/2, all (m,n) with n ≤ m
Active pairs	9	Pairs where m + n ≤ K
Inactive pairs	12	m + n > K; kernel ≈ 0, fitted hB ≈ 0

Fig. S 8 overlays the admissible triads on the empirical bispectrum map, and Fig. S 9 displays the K^3 → pairs → pairs+sym parameter reduction.

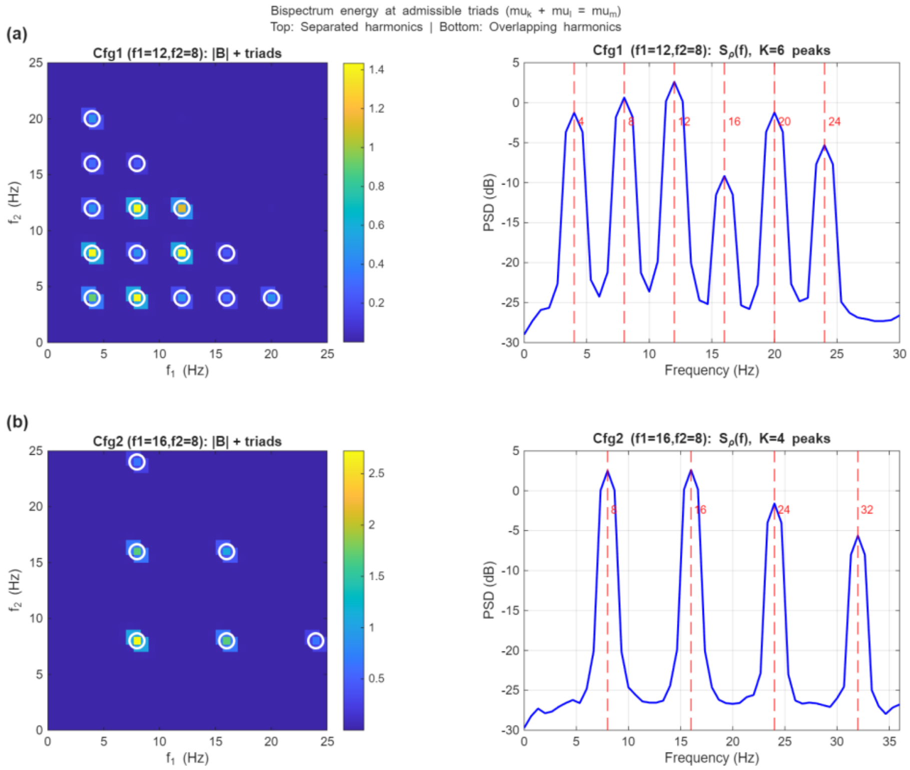

Fig. S 8. Bispectrum with admissible triad markers. *Bispectrum with admissible triad markers. Admissible triads on the closure manifold are overlaid on the bispectrum map to show which peak pairs satisfy the harmonic closure rule and therefore contribute to the BiSCA parametrization. The figure visualizes the sparse set of interactions retained by the closure-constrained model relative to the dense set of formally possible triads.*

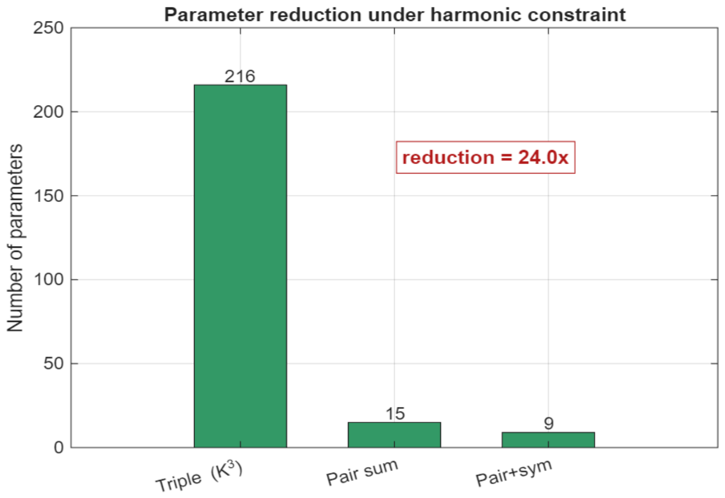

Fig. S 9. Parameter reduction under harmonic constraint. *The closure condition reduces the number of candidate bispectral interactions from the full* K^3 *enumeration to a much smaller set of admissible triads and unique peak pairs. The figure illustrates how harmonic organization converts a dense parameter search into a sparse, structurally constrained representation.*

Empirical verification in 3D frequency space. The preceding analysis relies on the algebraic closure condition m + n = k. To verify this independently, the full three-dimensional bispectral field $|E[X(f_1)\,X(f_2)\,X^*(f_3)]|$ is computed on a (f_1, f_2, f_3) grid without constraining $f_3 = f_1 + f_2$.

For a stationary process, $E[X(f_1)\,X(f_2)\,X^*(f_3)] = C_3^x(f_1, f_2) \cdot \delta(f_1 + f_2 - f_3)$: off-manifold values are theoretically zero. With finite data, off-manifold contributions have random phase across segments and cancel through averaging, while on-manifold contributions maintain coherent phase and accumulate.

Fig. S 10 shows the result for a simulation with K = 6 stochastic narrowband peaks at the harmonic frequencies from Table S.10. The bispectral energy (red/orange scatter) concentrates on the closure plane $f_3 = f_1 + f_2$ (blue surface), confirming the dimensionality reduction from 3D to 2D. Within the closure plane, energy localizes at the 15 admissible triad positions (green diamonds), confirming the further reduction from a continuous 2D surface to a discrete set of points determined by m + n = k. The grey cube $[0, F_s/2]^3$ delineates the full Nyquist-limited frequency space.

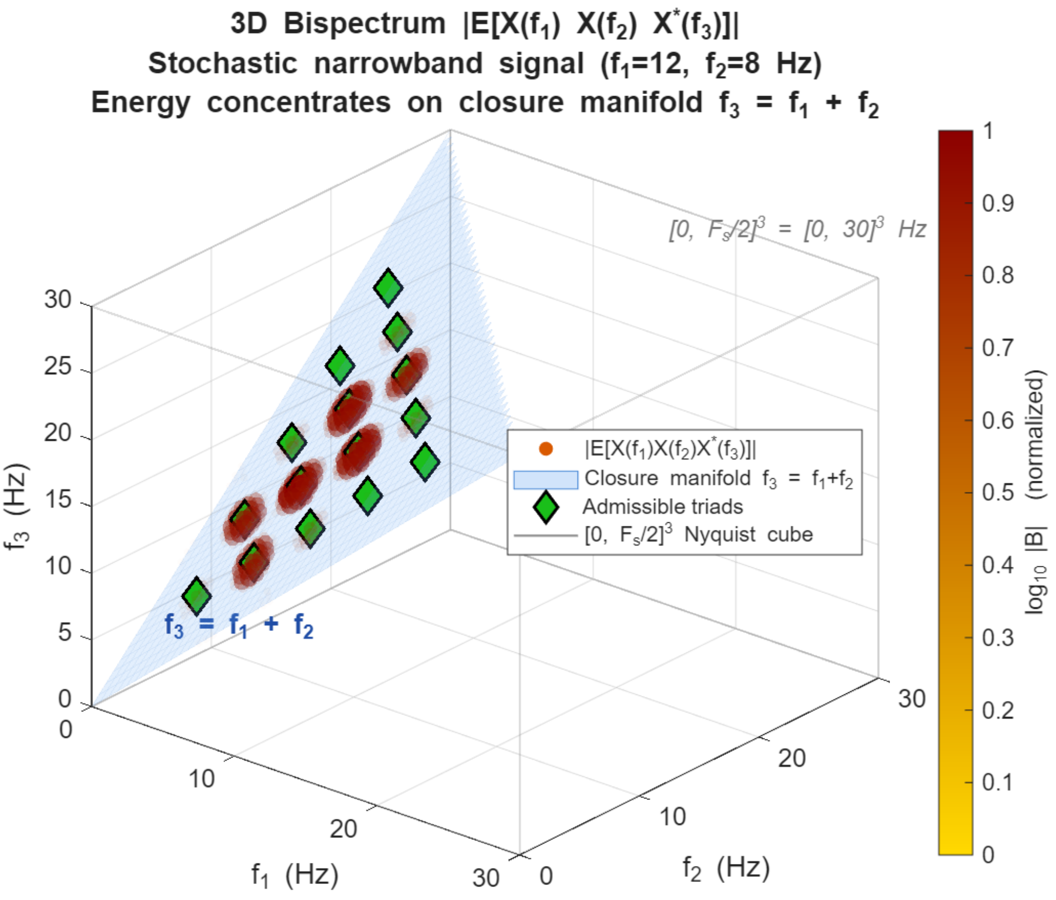

Fig. S 10. Empirical 3D bispectral field $|E[X(f_1)\, X(f_2)\, X^*(f_3)]|$ computed on a full (f_1, f_2, f_3) grid without constraining $f_3 = f_1 + f_2$. Signal: K = 6 stochastic narrowband peaks at 4, 8, 12, 16, 20, 24 Hz with quadratic coupling ($\alpha = 0.8$, $F_s = 60$ Hz, >7000 segments). Grey cube: Nyquist-limited space $[0, F_s/2]^3$. Blue surface: closure manifold $f_3 = f_1 + f_2$. Green diamonds: the 15 admissible triads from Table S.10. Red/orange scatter: empirical bispectral energy above the 99.5-th percentile threshold.

Pair count and reduction chain. The K(K+1)/2 formal pairs in Eq. S.1 enumerate all unordered pairs drawn from K component indices with replacement (self-pairs (m, m) included):

$$\binom{K+1}{2} = \underbrace{K}_{\text{self-pairs } (m,m)} + \underbrace{\binom{K}{2}}_{\text{cross-pairs } (m,n),\ m>n} = \frac{K(K+1)}{2}$$

For K = 6: 6 self-pairs + 15 cross-pairs = 21 formal pairs. The 15 admissible triads reduce to 9 unique pairs after permutation symmetry; thus 15 → 9 ⊂ 21, with 21 = 9 + 12. Eq. S.1 includes the 12 non-admissible pairs (m + n > K) by design: for these, the product kernel $t_2(f_1, f_2; \mu_m, \mu_n)$ is negligible everywhere because the third factor is centered beyond the last spectral peak, and the fitted hB values converge to approximately zero. Retaining all formal pairs also allows the model to capture near-harmonic interactions when peak centers are fitted freely.

Table S 13. The three counts for K = 6.

Count	What it counts	Origin
15	Admissible triads, both orders of (m,n)	Closure m+n = k, all orderings
9	Unique admissible pairs with n ≤ m and m+n ≤ K	Closure + permutation symmetry
21	Formal pairs in the double sum of Eq. S.1	K(K+1)/2: all pairs with replacement

Assembling all steps, the full reduction proceeds as follows. Starting from the general triple-index expansion with K^3 terms,

$$\sum_{m=1}^{K}\sum_{n=1}^{K}\sum_{k=1}^{K} \mathbb{E}\left[X_{\rho,m}(f_1)\, X_{\rho,n}(f_2)\, X^*_{\rho,k}(f_1+f_2)\right]$$

the narrowband closure condition ($\mu_m + \mu_n \approx \mu_k$) retains only triads where all three kernels overlap. Under harmonicity (m + n = k), the index k is uniquely determined, collapsing the triple sum to a double

sum. Permutation symmetry $B(f_1, f_2) = B(f_2, f_1)$ identifies pairs (m, n) ~ (n, m), restricting to n ≤ m. For each admissible pair, the phase and amplitude factors collapse to a single complex scalar:

$$h_{B,m,n} := h_{\rho,m}^{1/2}\, h_{\rho,n}^{1/2}\, h_{\rho,k}^{1/2}\; e^{i(\phi_m+\phi_n-\phi_k)}$$

where the biphase $\varphi_m + \varphi_n - \varphi_k$ is absorbed into $\arg(h_{B,m,n})$ and the amplitude product into $|h_{B,m,n}|$ (standard narrowband approximation). Extending the sum to all K(K+1)/2 formal pairs for implementation generality yields Eq. S.1; the kernel automatically suppresses non-admissible terms. Adding the aperiodic term $h_{B,\xi,\xi}\, t_2(f_1, f_2; \mu_\xi, \mu_\xi, \sigma_\xi, \nu_\xi, d_\xi)$ completes Eq. (15) of the main text.

General admissible pair count. For K harmonic peaks with $\mu_m = m f_0$, the number of closure-admissible pairs (m, n) with n ≤ m and m + n ≤ K is:

$$N_{\text{pairs}} = \lfloor K^2/4 \rfloor$$

Table S 14. Admissible pair counts vs. formal pair counts.

K	K^3 (triple)	Admissible triads	Admissible pairs	K(K+1)/2 (formal)
2	8	1	1	3
3	27	3	2	6
4	64	6	4	10
5	125	10	6	15
6	216	15	9	21

The narrowband closure condition assumes each component occupies a narrow spectral band. Broadband cross-frequency interactions—such as theta–gamma phase-amplitude coupling, which produces extended ridges rather than localized peaks in the bicoherence—are not captured by the present pair-indexed model. Modeling such interactions would require frequency-dependent quadratic kernels $H_2(\omega_1, \omega_2)$ beyond the separable structure assumed here.

S.6 Theoretical Derivation and Modeling Assumptions

System linearity/nonlinearity and input Gaussianity/non-Gaussianity are orthogonal properties and should not be conflated. A linear system can produce non-Gaussian output when driven by non-Gaussian i.i.d. innovation. A nonlinear system can produce approximately Gaussian output under aggregation (central-limit effects). In the Brillinger-Hinich setting, input non-Gaussianity is characterized by the third cumulant μ_3, while system nonlinearity is characterized by higher-order Volterra kernels ($H_2, H_3, \ldots$). These mechanisms produce distinct bicoherence signatures: constant bicoherence across frequencies for linear systems with non-Gaussian i.i.d. input, versus frequency-structured peaks for nonlinear phase coupling. Power-spectrum inspection alone cannot disambiguate these generative mechanisms; higher-order spectral analysis is required.

S.6.1 Generative framework: linear and nonlinear process models

Linear process: Wold decomposition

Wold's Theorem: Any stationary, zero-mean time series can be decomposed as:

$$X_t = \underbrace{\sum_{j=0}^{\infty} a_j \epsilon_{t-j}}_{MA\,(\infty)\ component} + D_t$$

where ϵ_t is an i.i.d. innovation and D_t is a deterministic component (typically zero for stochastic processes). Representative finite-order linear process forms are summarized in Table S15.

Table S 15. Finite-order approximations:

Form	Time Domain	Transfer Function $H(\omega)$
AR(p)	$X_t = \sum_{k=1}^{p} \phi_k X_{t-k} + \epsilon_t$	$\frac{1}{1 - \sum_{k=1}^{p} \phi_k e^{-i\omega k}}$
MA(q)	$X_t = \sum_{j=0}^{q} \theta_j \epsilon_{t-j}$	$\sum_{j=0}^{q} \theta_j e^{-i\omega j}$
ARMA (p, q)	$X_t - \sum_{k} \phi_k X_{t-k} = \sum_{j} \theta_j \epsilon_{t-j}$	$\frac{\sum_{j=0}^{q} \theta_j e^{-i\omega j}}{1 - \sum_{k=1}^{p} \phi_k e^{-i\omega k}}$

Key insight: AR, MA, and ARMA are equivalent representations of the same linear process class—they differ only in parameterization, not in the underlying stochastic structure.

i.i.d. Innovation requirements: $E[\epsilon_t] = 0$ (zero mean), $\mathrm{Var}(\epsilon_t) = \sigma^2$ (constant variance) $\epsilon_t \perp \epsilon_s$ for $t \neq s$ (independence), $E[\epsilon_t^3] = \mu_3$ (third cumulant; = 0 for Gaussian), $\mathrm{cum}(\epsilon_t, \epsilon_s, \epsilon_r) = \mu_3 \cdot \delta_{t,s,r}$ (diagonal structure due to i.i.d.)

Nonlinear process: Volterra series

The Volterra functional series generalizes linear convolution to nonlinear systems. Any fading-memory nonlinear system can be expanded as:

$$X_t = \underbrace{\sum_j h_1(j)\,\epsilon_{t-j}}_{Linear\ (1st\ order)} + \underbrace{\sum_{j,k} h_2(j,k)\,\epsilon_{t-j}\epsilon_{t-k}}_{Quadratic\ (2nd\ order)} + \cdots$$

where $h_p(\tau_1, \ldots, \tau_p)$ is the p-th order Volterra kernel and ϵ_t is the i.i.d. innovation.

In frequency domain:

$$X(\omega) = H_1(\omega)U(\omega) + \iint H_2(\omega_1, \omega_2)U(\omega_1)U(\omega_2)\delta(\omega_1 + \omega_2 - \omega)\,d\omega_1\,d\omega_2 + \cdots$$

Special structures of H_2: Wiener model (linear filter to static nonlinearity): $H_2(\omega_1, \omega_2) = \alpha \cdot H_1(\omega_1)H_1(\omega_2)$ separable; used in BiSCA (Marzocca et al., 2008)[41]. General Volterra: $H_2(\omega_1, \omega_2)$ non-separable more expressive but harder to identify from data

Spectral consequences: H_1: determines the power spectrum shape ($S_X = |H_1|^2\sigma^2$ at leading order) H_2: introduces quadratic phase coupling (QPC), creating bispectral peaks at triads $(f_1, f_2, f_1 + f_2)$. Higher kernels $H_3, \ldots$: contribute to trispectrum and beyond (not modeled in BiSCA).

The retained-kernel truncations and their spectral consequences are summarized in Table S 16.

Table S 16 Truncated approximations (by kernel order)

Truncation	Retained Kernels	Spectral Consequence
Linear ($p = 1$)	h_1 only	Reduces to Wold representation (Section S.6.1)
Quadratic ($p \leq 2$)	h_1, h_2	Bispectral peaks from quadratic phase coupling; BiSCA operates here
Cubic ($p \leq 3$)	h_1, h_2, h_3	Adds trispectral structure (not modeled in BiSCA)

Key insight: The linear case (Section S.6.1) is the special case $h_2 = h_3 = \cdots = 0$. Bispectral analysis specifically targets the quadratic kernel h_2.

S.6.2 Spectral implications of the four generative classes

$$S_X(\omega) = \sum_{\tau=-\infty}^{\infty} R_X(\tau) e^{-i\omega\tau} \quad \text{where } R_X(\tau) = E[X_t X_{t+\tau}]$$

For linear systems with i.i.d. input: $S_X(\omega) = |H(\omega)|^2 \cdot \sigma^2$

Bispectrum:

$$B_X(\omega_1, \omega_2) = E[X(\omega_1)X(\omega_2)X^*(\omega_1 + \omega_2)]$$

Power spectrum:

$$f_X(\omega) = E[|X(\omega)|^2]$$

Squared bicoherence:

$$|b(\omega_1, \omega_2)|^2 = \frac{|B_X(\omega_1, \omega_2)|^2}{f_X(\omega_1) f_X(\omega_2) f_X(\omega_1 + \omega_2)}$$

Case 1: Linear + Gaussian i.i.d. (GL)

System: Linear (AR, MA, or ARMA — all equivalent by Wold) and Innovation: $\epsilon_t \sim N(0, \sigma^2)$, i.i.d..

Step 1 — Gaussian third cumulant: For Gaussian random variables, all cumulants of order > 2 are zero:

$$\mu_3 = E[\epsilon_t^3] = 0$$

Step 2 — Bispectrum of linear process: For any linear process $X(\omega) = H(\omega) \cdot U(\omega)$ with i.i.d. input (Brillinger 2001[45], Examples 2.9.1-2.9.2):

$$B_X(\omega_1, \omega_2) = \mu_3 \cdot H(\omega_1) \cdot H(\omega_2) \cdot H^*(\omega_1 + \omega_2)$$

Step 3 — Result: Substituting $\mu_3 = 0$:

$$B_X(\omega_1, \omega_2) = 0$$

Therefore, taking the square root:

$$|b(\omega_1, \omega_2)| = 0 \quad \text{(GL)}$$

Case 2: Linear + Non-Gaussian i.i.d. (NGL)

System: Linear (AR, MA, or ARMA — all equivalent by Wold) and Innovation: ϵ_t is i.i.d. with non-Gaussian distribution (e.g., Pearson Type III) $\mu_3 = E[\epsilon_t^3] \neq 0$.

Step 1 — Bispectrum:

$$B_X(\omega_1, \omega_2) = \mu_3 \cdot H(\omega_1) \cdot H(\omega_2) \cdot H^*(\omega_1 + \omega_2)$$

Step 2 — Power spectrum:

$$S_X(\omega) = \sigma^2 |H(\omega)|^2$$

Step 3 — Bicoherence (key cancellation):

$$|b|^2 = \frac{|B_X|^2}{S_X(\omega_1) \cdot S_X(\omega_2) \cdot S_X(\omega_1 + \omega_2)}$$

$$= \frac{|\mu_3|^2 |H(\omega_1)|^2 |H(\omega_2)|^2 |H(\omega_1 + \omega_2)|^2}{\sigma^6 |H(\omega_1)|^2 |H(\omega_2)|^2 |H(\omega_1 + \omega_2)|^2} = \frac{\mu_3^2}{\sigma^6}$$

The $|H(\omega)|^2$ terms cancel completely. Taking the square root:

$$|b(\omega_1, \omega_2)| = |\gamma_3| = |\mu_3|/\sigma^3 = \text{CONSTANT} \quad \text{(NGL)}$$

This is Berg et al. (2010, p.5)[46]: “if a time series is linear, its normalized bispectrum is constant over all (λ_1, λ_2)”

The transfer function $H(\omega)$ shapes the power spectrum (giving peaks at resonances), but this frequency dependence is normalized out in bicoherence. What remains is only the input’s skewness μ_3/σ^3. This distinction is useful in practice because the bispectral numerator and denominator may each retain strong frequency structure while their ratio remains approximately constant for an NGL process. This behavior is illustrated in Fig. S11 for an NGL simulation (AR(1) $a = 0.95$, Pearson-III innovation, $T = 180$ s).

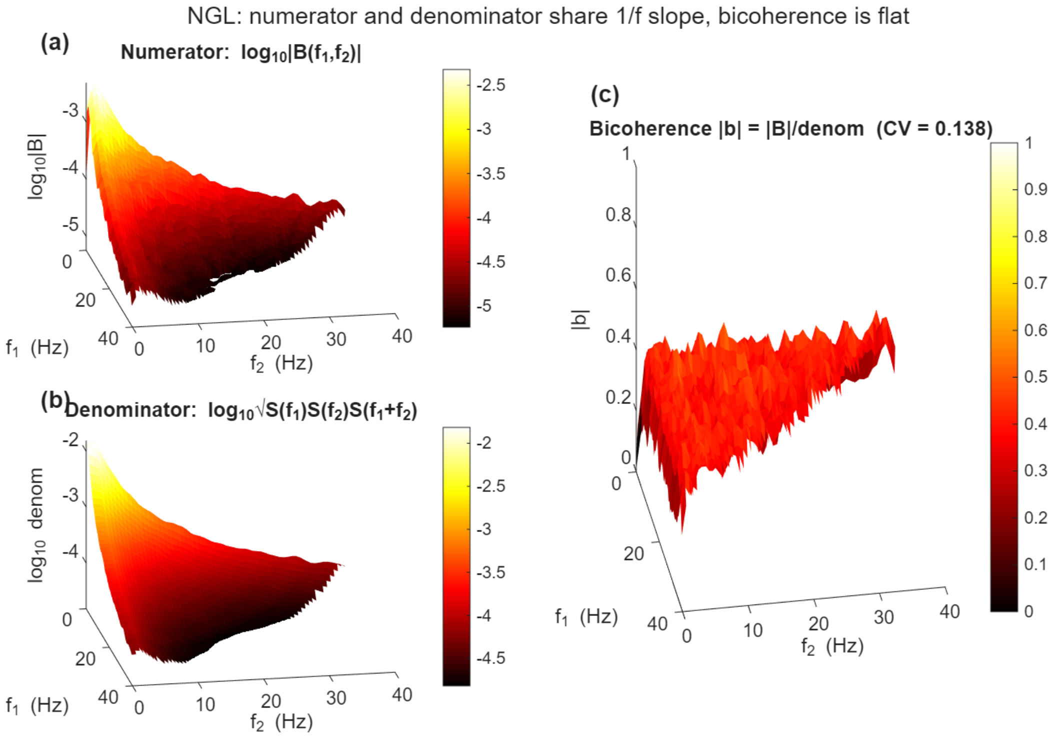

Fig. S 11. Bicoherence normalization in a linear non-Gaussian (NGL) process. *a, magnitude of the bispectral numerator, $|B(f_1, f_2)|$. b, magnitude of the normalization denominator, $[S(f_1)S(f_2)S(f_1 + f_2)]^{1/2}$. Both are shown on a log_{10} scale. c, resulting bicoherence magnitude, $|b(f_1, f_2)| = |B(f_1, f_2)|/[S(f_1)S(f_2)S(f_1 + f_2)]^{1/2}$. The numerator and denominator retain similar frequency-dependent structure imposed by the linear filter, but this shared scaling is removed by normalization, leaving an approximately flat bicoherence surface. The figure therefore illustrates why near-constant bicoherence in the NGL case does not imply a trivial bispectral numerator.*

Case 3: Nonlinear + Gaussian i.i.d. (GNL)

System: Nonlinear (Volterra with H_2 kernel, e.g., NAR: $X_t = \phi X_{t-1} + \beta X_{t-1}^2 + \epsilon_t$) and innovation: $\epsilon_t \sim N(0, \sigma^2)$, i.i.d..For a Volterra system, the output bispectrum has contributions from both H_1 (linear) and H_2 (quadratic):

$$B_X(\omega_1,\omega_2) = \underbrace{\mu_3 \cdot H_1(\omega_1) H_1(\omega_2) H_1^*(\omega_1+\omega_2)}_{Linear\ contribution\ (=\ 0\ for\ Gaussian)} + \underbrace{B_{H_2}(\omega_1,\omega_2)}_{Nonlinear\ contribution}$$

For Gaussian input, $\mu_3 = 0$, so the linear contribution vanishes. But the nonlinear kernel H_2 creates quadratic phase coupling (QPC). The leading interaction term is (Nichols et al. 2009):

$$B_{H_2}(\omega_1, \omega_2) \approx 2\sigma^4 \cdot H_1(\omega_1)H_1(\omega_2) \cdot H_2^*(\omega_1, \omega_2)$$

Peaks arise because: $H_2^*(\omega_1, \omega_2)$ doesn't factor as $H \cdot H \cdot H^*$ and normalization doesn't cancel frequency dependence. Result: $|b(\omega_1, \omega_2)|$ has peaks at frequencies where $H_2(\omega_1, \omega_2)$ is large

$$|b(\omega_1, \omega_2)| = \text{frequency-dependent (peaks)} \qquad \text{(GNL)}$$

The nonlinear kernel creates energy transfer between frequencies (e.g., $\alpha + \alpha \rightarrow 2\alpha$ frequency doubling), which manifests as localized peaks in bicoherence at harmonic frequencies.

Case 4: Nonlinear + Non-Gaussian i.i.d. (NGNL)

System: Nonlinear (Volterra with H_2 kernel) and Innovation: ϵ_t is i.i.d. with non-Gaussian distribution, $\mu_3 \neq 0$. The bispectrum now has both contributions:

$$B_X(\omega_1,\omega_2) = \underbrace{\mu_3 \cdot H_1 \cdot H_1 \cdot H_1^*}_{Non-Gaussian\ \ contribution} + \underbrace{B_{H_2}(\omega_1,\omega_2)}_{Nonlinear\ \ contribution}$$

After normalization: The first term gives a constant background (like NGL case): $|\gamma_3|$. The second term gives frequency-dependent peaks (like GNL case): $f(H_2)$

Result: To leading order, bicoherence has peaks superimposed on a non-zero background:

$$|b(\omega_1, \omega_2)| \approx |\gamma_3| + f(H_2) = \text{constant background} + \text{peaks} \quad \text{(NGNL)}$$

Colored noise and the cascade property

If $\epsilon_t = \sum_k b_k \, w_{t-k}$ (linear filter of i.i.d. w_t) and $X_t = \sum_j a_j \, \epsilon_{t-j}$ (second linear filter), then:

$$X_t = \sum_j a_j \sum_k b_k \, w_{t-j-k} = \sum_m c_m \, w_{t-m}, \quad c_m = (a * b)_m$$

In the frequency domain: $H_{\text{total}}(\omega) = G_1(\omega) \cdot G_2(\omega)$, where G_1 and G_2 are the transfer functions of the two linear stages. The cascade reduces to a single linear filter of the original i.i.d. source w_t, so the constant-bicoherence theorem applies regardless of how many linear filtering stages intervene.

The trivial model $y(t) = x(t)$

Setting $y(t) = x(t)$ with $A(\omega) = 1$ gives bispectrum equal to that of the structured signal itself:

$$B_X(\omega_1, \omega_2) = B_x(\omega_1, \omega_2)$$

$$|b(\omega_1, \omega_2)|^2 = \frac{|B_x(\omega_1, \omega_2)|^2}{f_x(\omega_1) \, f_x(\omega_2) \, f_x(\omega_1 + \omega_2)} \neq \text{constant}$$

Since $x(t)$ carries temporal structure and is not i.i.d., the factorization $B_X = \mu_3 HHH^*$ (Eq. 8) does not hold, and the $|H|^2$ cancellation that produces constant bicoherence does not occur. This construction falls outside the definition of a linear process rather than contradicting the theorem.

S.6.3 Additive decomposition and the independence assumption

We note that x_ξ and x_ρ is a commonly used modeling assumption in periodic/aperiodic decomposition, not unique to our method. We treat this independence primarily as a working assumption for cumulant additivity (Brillinger, 2001, Chapter 4)[45], informed by mathematical proof, neurophysiological considerations, and numerical validation.

Under the assumptionx_ξ and $x_\rho$are independent, cumulants of all orders decompose additively (Brillinger, 1965, Eq. 4.11; Nikias & Petropulu, 1993, Properties 3–4, pp. 13–14)[42,25],

$$\text{cum}_k(x) = \text{cum}_k(x_\xi) + \text{cum}_k(x_\rho), \quad \forall k,$$

which implies

$$S_x = S_\xi + S_\rho (k = 2)\,, \qquad B_x = B_\xi + B_\rho \ (k = 3).$$

The BiSCA bispectrum model (Eq. 15) retains both B_ξ and B_ρ as explicit terms with free complex amplitudes ($h_{B,\xi}$ and $h_{B,\rho,m,n}$). If ξ is Gaussian linear (GL), $B_\xi = 0$ and $h_{B,\xi} = 0$; if ξ is non-Gaussian linear (NGL), $h_{B,\xi} \neq 0$ and the model captures the flat bispectral background. The empirical finding $h_{B,\xi} \approx 0$ across both datasets provides data-driven support for the GL interpretation, so the bispectral structure is attributed to x_ρ. The spectrum proof follows from the vanishing of cross-spectrum terms under independence, and the bispectrum proof follows because all mixed third-order cumulants vanish.

For spectrum we have $S_x = E[(X_\xi + X_\rho)(X_\xi^* + X_\rho^*)] = S_\xi + S_{\xi\rho} + S_{\rho\xi} + S_\rho$. Independence ⇒ $E[x_\xi(t)x_\rho(s)] = 0$ for all t, s, so $S_{\xi\rho} = S_{\rho\xi} = 0$ and $S_x = S_\xi + S_\rho$. For bispectrum, expanding $X = X_\xi + X_\rho$ in $B_x = E[X(f_1)X(f_2)X^*(f_1 + f_2)]$ yields $2^3 = 8$ terms: two pure (B_ξ, B_ρ) and six mixed. Each mixed term is a mixed 3rd-order cumulant that vanishes under independence. Hence $B_x = B_\xi + B_\rho$; the fitted value $h_{B,\xi} \approx 0$ yields $B_x \approx B_\rho$ (see S.6.4 for the dynamical-system interpretation).

Oscillatory generators can be lamina-specific [47], with distinct laminar directionality for low- vs. high-frequency rhythms[48] and a reported spectrolaminar motif [49]. The aperiodic trend may arise partly from subcritical network dynamics whose changes affect peaks and trend relatively independently; however,

shared biophysical parameters (synaptic kinetics, E/I ratio) multiplicatively affect both components, so full statistical independence is not guaranteed [50]. Our framework adopts an additive periodic/aperiodic decomposition on the natural power scale. In this respect it is closer in spirit to ξ-π and to IRASA, which also separate periodic and aperiodic structure on the natural spectrum scale, than to log-spectrum parameterizations such as specparam. At the same time, these approaches still differ in parameterization, estimation procedure and inferential scope. Definitive verification requires experimental designs that can isolate the two components at the source level. Periodic and aperiodic parameters exhibit differential sensitivity to disease (Alzheimer[51]), pharmacology (haloperidol[52]), and developmental trajectories (distinct lifespan curves that may reflect different biological substrates[53]).

The assumption is also supported numerically Fig. S 12. Two simulation configurations (shared setup: x_ξ = AR(1) $a = 0.98$, quadratic nonlinearity $\alpha = 0.8$, $T = 60$ s, $F_s = 200$ Hz)—Config 1 ($f_1 = 12$, $f_2 = 8$ Hz, separated harmonics) and Config 2 ($f_1 = 16$, $f_2 = 8$ Hz, overlapping harmonics)—support the following: (i) same-frequency cross-spectra are negligible within ρ (off/diag < -33 dB; Figure S.12), (ii) composite-level additivity approximately holds ($S_x \approx S_\xi + S_\rho$; spectrum error $\leq 1.1\%$, bispectrum error $\leq 10.3\%$; Figure S.13). The bispectral error is consistent with the higher estimation variance of 3rd-order statistics[45], rather than clear evidence of Xi-Rho leakage.

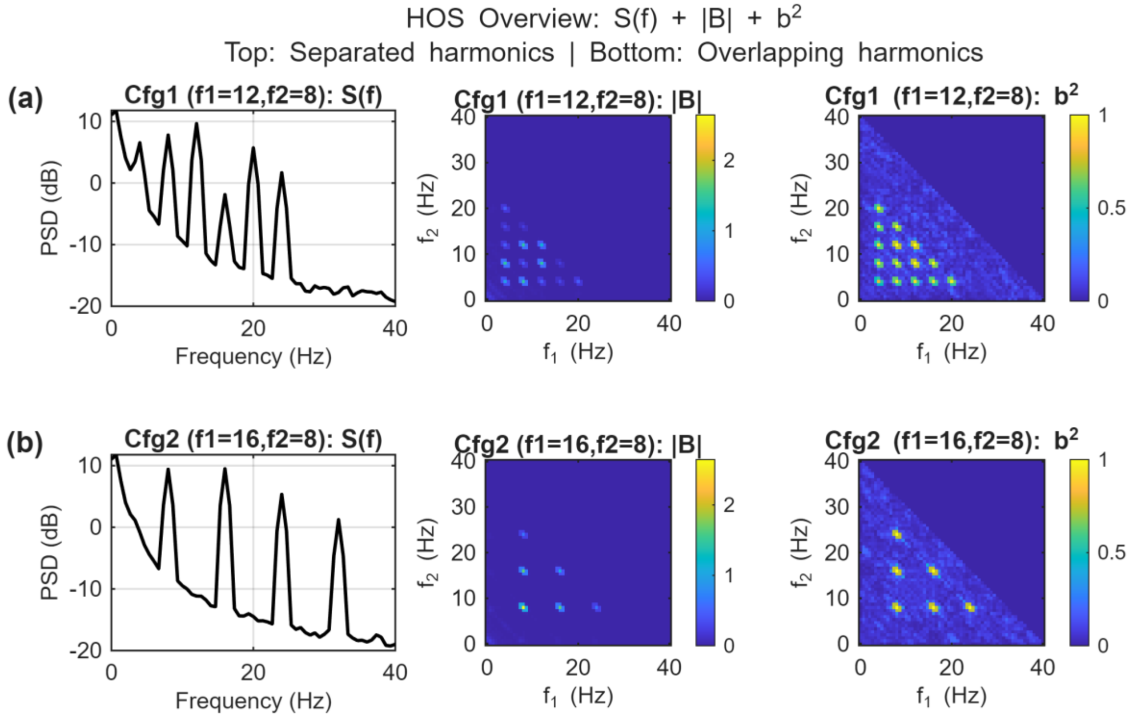

Fig. S 12. *Higher-order spectral overview for the two simulation configurations used in the numerical validation of Section S.6.3. a, configuration 1 ($f_1 = 12$ Hz, $f_2 = 8$ Hz), in which the harmonic peaks are separated. b, configuration 2 ($f_1 = 16$ Hz, $f_2 = 8$ Hz), in which harmonic peaks overlap. For each configuration, left to right show the power spectral density $S(f)$, the bispectrum magnitude $|B|$, and the squared bicoherence b^2. Although harmonic overlap reduces spectral separability in $S(f)$, the higher-order spectra retain localized coupling hotspots in both configurations, illustrating that bispectral structure remains detectable under both separated- and overlapping-harmonic conditions.*

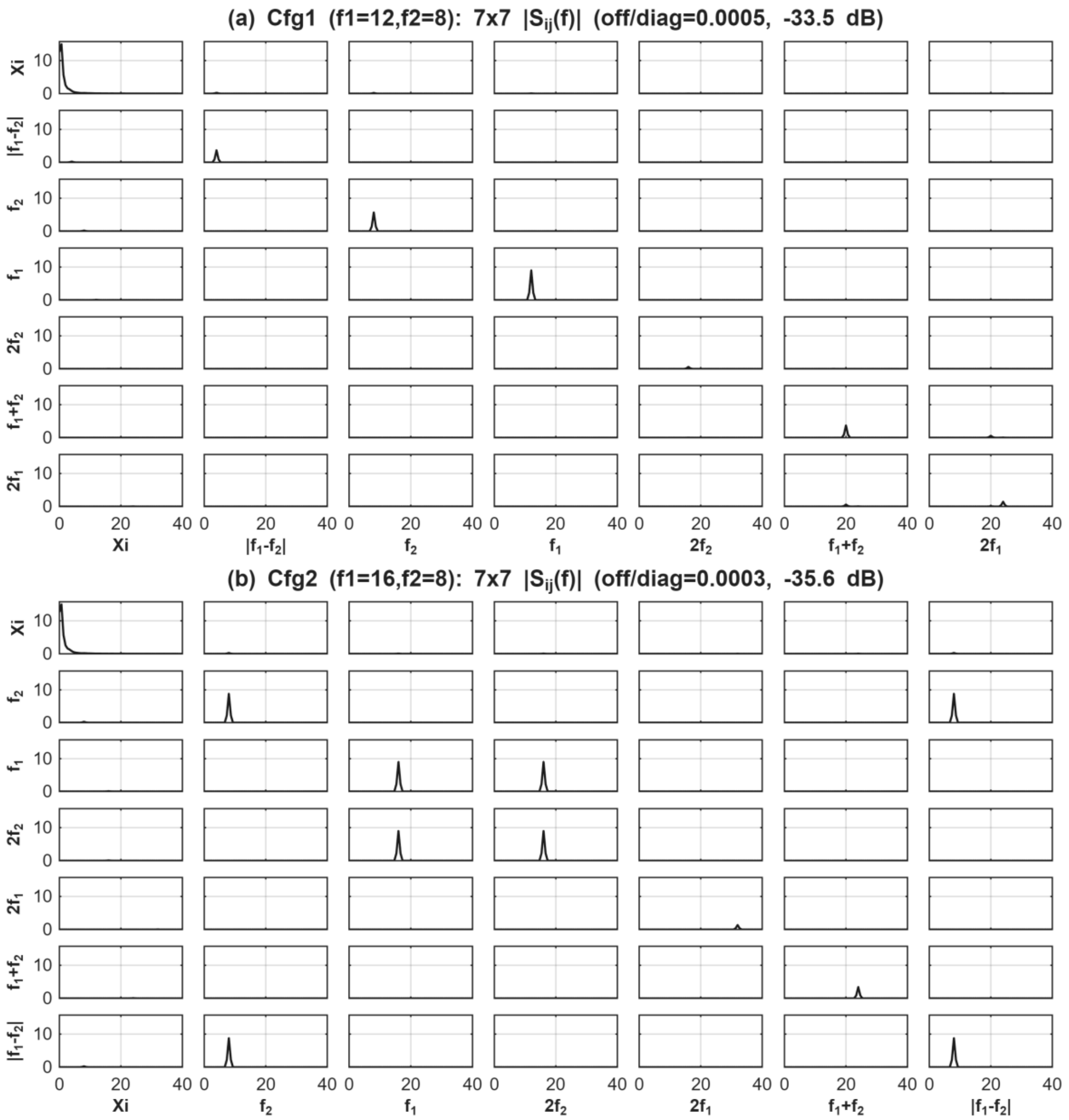

Fig. S 13. *Cross-spectrum grid within the oscillatory component rho. Same-frequency cross-spectrum magnitude grid for the simulation components used to assess additive decomposition. a, configuration 1 ($f_1 = 12$ Hz, $f_2 = 8$ Hz; separated harmonics). b, configuration 2 ($f_1 = 16$ Hz, $f_2 = 8$ Hz; overlapping harmonics). Each 7×7 matrix shows $|S_{ij}(f)|$ for ξ and the peak-centered narrowband signals associated with the oscillatory peaks. In both configurations, spectral energy is concentrated on the diagonal auto-spectral terms, whereas off-diagonal cross-spectra remain markedly smaller (median off-diagonal/diagonal ratios 0.0005 and 0.0003, corresponding to -33.5 dB and -35.6 dB), consistent with near-diagonal same-frequency second-order structure despite the presence of bispectral coupling.*

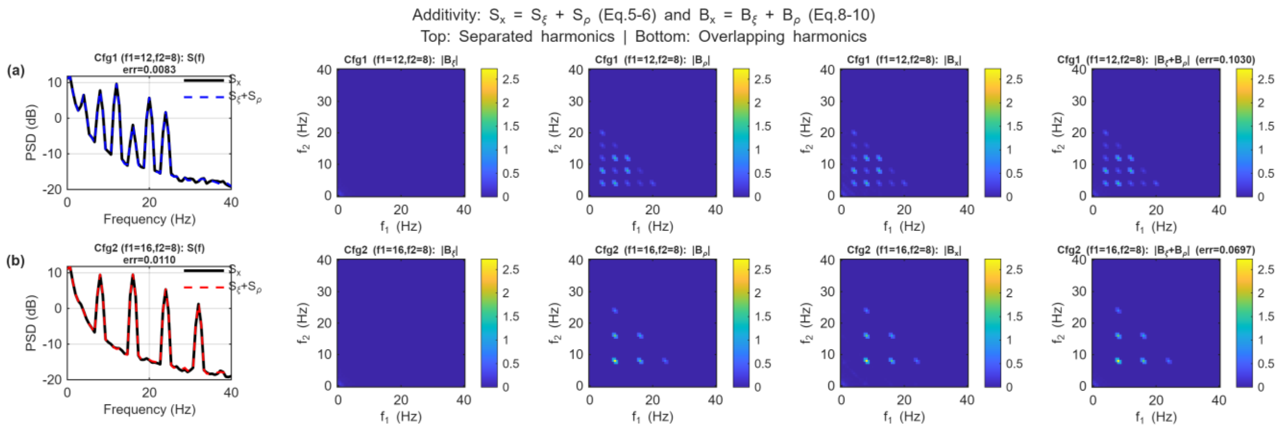

Fig. S 14. *Additivity verification. Numerical validation of additive spectral and bispectral decomposition. a, configuration 1 ($f_1 = 12$ Hz, $f_2 = 8$ Hz; separated harmonics). b, configuration 2 ($f_1 = 16$ Hz, $f_2 = 8$ Hz; overlapping harmonics). In each row, the left panel shows the composite power spectrum S_x (black) and the component sum $S_\xi + S_\rho$ (dashed), and the four right panels show $|B_\xi|$, $|B_\rho|$, $|B_x|$ and $|B_\xi + B_\rho|$ on a shared color scale. Across both configurations, $|B_\xi|$ is negligible and $|B_x|$ closely matches $|B_\xi + B_\rho|$, supporting the approximate additive relations $S_x \approx S_\xi + S_\rho$ and $B_x \approx B_\xi + B_\rho$. Error values denote normalized L_2 mismatches: 0.0083 and 0.0110 for the spectra, and 0.1030 and 0.0697 for the bispectra, for panels a and b, respectively.*

Example of BiSCA model adequacy for the supplementary validation signal is shown in Fig. S15, where the empirical bispectrum, the model-reconstructed bispectrum, and the residual are compared side by side for both magnitude and phase.

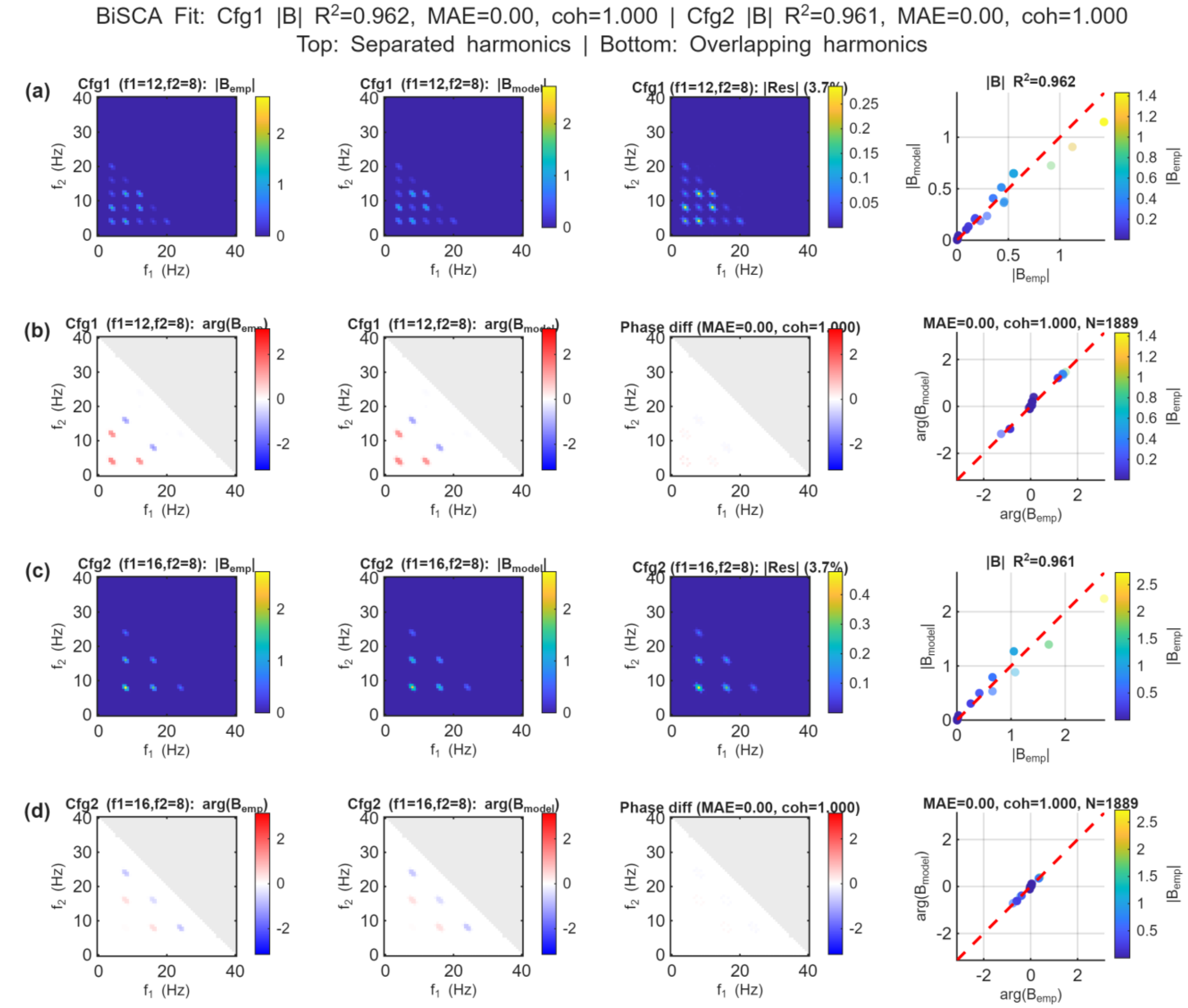

Fig. S 15. *BiSCA bispectrum model fit to synthetic signal (supplementary validation, Analysis C). Left: empirical bispectrum magnitude* $|B_{emp}|$ *showing the main harmonic-coupling peaks. Center: model-reconstructed bispectrum magnitude* $|B_{model}|$ *after optimal complex scaling. Right: residual* $|B_{emp} - B_{model}|$*. The spectrum fit is* $R_S^2 = 99.9\%$ *, the bispectrum fit is* $R_B^2 = 94.4\%$ *, and the residual bispectral energy is 5.3%.*

Periodic/aperiodic separation is a working assumption; perfect separation is not achievable given the infinite solutions to this inverse problem[54]. The Xi and Alpha independence assumption likewise warrants further empirical investigation across brain regions, states, and pathologies.

S.6.4 Kernel function and dynamical-system interpretation

The generalized Student-t kernel used in BiSCA (Eq. 14) admits a direct dynamical-system interpretation in the Lorentzian special case. When $\nu = 1$ and $d = 2$, the kernel reduces to the Cauchy–Lorentz form

$$t(f;\ \mu, \sigma, 1{,}2) = \frac{1}{1 + \left(\frac{f-\mu}{\sigma}\right)^2}$$

which is the squared modulus of the first-order transfer function $|H_1(f)|^2$ of a linear stochastic differential equation driven by stationary noise. For the aperiodic component ($\mu = 0$), the transfer function is $H_1(f) = (1 + i2\pi f/\lambda)^{-1}$ with $\lambda = 2\pi\sigma_\xi$, corresponding to an Ornstein–Uhlenbeck (OU) process—a continuous-time AR(1) with exponentially decaying autocorrelation $R_\xi(\tau) \propto e^{-\lambda|\tau|}$. This is the canonical model for noise-driven dynamics linearized near a stable equilibrium; its power spectrum is a Lorentzian with a characteristic knee at σ_ξ, flat at low frequencies and rolling off above the knee. For each oscillatory component ($\mu = \mu_{\rho,k} > 0$), the transfer function becomes $H_1(f) = (1 + i2\pi(f - \mu_{\rho,k})/\lambda_k)^{-1}$, corresponding to a damped harmonic oscillator—equivalently, a continuous-time AR(2) with complex-conjugate poles—whose autocorrelation is a damped cosine, $R_{\rho,k}(\tau) \propto e^{-\lambda_k|\tau|}\cos(2\pi\mu_{\rho,k}\tau)$, where $\lambda_k = 2\pi\sigma_{\rho,k}$ is the damping rate and $\mu_{\rho,k}$ the natural frequency.

Crucially, the Lorentzian spectral shape characterizes the linear transfer function $|H_1(f)|$ and is independent of whether the driving noise is Gaussian or non-Gaussian. If the input noise has a nonzero third-order cumulant $\gamma_3 \neq 0$ (non-Gaussian linear, NGL), the power spectrum remains Lorentzian—determined entirely by H_1—while the bispectrum becomes $B_\xi(f_1, f_2) = H_1(f_1)\, H_1(f_2)\, H_1^*(f_1 + f_2)\, \gamma_3$, producing a flat nonzero bicoherence $|b_\xi| = |\gamma_3|/\sigma^3 = \text{const} > 0$. The BiSCA bispectrum model (Eq. 15) includes an explicit aperiodic term $h_{B,\xi}\, t_2(f_1, f_2; 0{,}0)$ with a free complex amplitude $h_{B,\xi} \in \mathbb{C}$, which absorbs γ_3 in the NGL case. The Gaussian linear (GL) interpretation—$B_\xi = 0$—is therefore not a structural constraint of the model but an empirical finding: the fitted value $h_{B,\xi} \approx 0$ across both datasets (consistent with the flat near-zero ξ bicoherence in Fig. 1F) provides data-driven support for the GL interpretation.

For general (ν, d), the kernel no longer corresponds to a single named dynamical system and the time-domain autocorrelation involves special functions that do not reduce to elementary forms. The parameter ν controls the tail decay rate: $\nu < 1$ produces heavier tails (broader frequency spread than a single-pole filter), while $\nu > 1$ yields sharper peaks (consistent with higher-order poles or resonance amplification). The parameter d is not an independent dynamical-system quantity but a methodological consequence of multitaper spectral smoothing: the finite bandwidth $2NW/T$ of the multitaper estimator broadens and flattens intrinsically Lorentzian peaks, and d absorbs this distortion so that the

remaining parameters (μ, σ, ν) retain their physical interpretation. In practice, fitted values of d are larger than 2, consistent with the smoothing introduced by the multitaper method (see Methods Section 4.2.4). The Lorentzian special case thus anchors the parametrization to well-understood stochastic processes, while the general kernel accommodates the empirical diversity of spectral shapes as observed through multitaper estimation. We thank an anonymous reviewer for prompting us to formalize this dynamical-system connection.

S.7 Complementary Approaches to the Linearity of Macroscopic Brain signals

Three recent methodologies address whether macroscopic brain signals are adequately described by linear Gaussian models. They operate in different statistical domains and are best regarded as complementary rather than competing tests. The relationship among these three complementary approaches is summarized in Table S17.

Table S 17 Methodologies

	Time domain	Information-theoretic	Frequency
Paper	Nozari et al. (2024)[55]	Tani Raffaelli et al. (2024)[56]	BiSCA (this work)
Statistic	Prediction R^2 (linear vs. nonlinear AR)	Relative Non-Linearity: $\mathrm{RNL} = 1 - \mathrm{MI}_{\mathrm{Gauss}}/\mathrm{MI}_{\mathrm{total}}$	Bicoherence $\hat{b}(f_1, f_2)$ tested against $\chi^2(2)$ null
Null hypothesis	Linear AR model sufficient	Gaussian copula sufficient	Linear Gaussian process without quadratic phase coupling
Frequency resolution	None	None	Full (f_1, f_2) bifrequency map
Separates nonlinearity from non-Gaussianity	No	No	Yes, within the quadratic-bicoherence setting
Data modalities	fMRI, iEEG	Spikes, iEEG, EEG, fMRI	iEEG, EEG

Time-domain prediction frameworks such as Nozari et al. (2024)[55] evaluate whether nonlinear predictors improve forecast accuracy over linear baselines. Their strength lies in addressing predictive sufficiency, but prediction R^2 is not intended to localize phase-coupled interactions in bifrequency space.

Information-theoretic frameworks such as Tani Raffaelli et al. (2024)[55] quantify dependence beyond a Gaussian-copula baseline. Their strength lies in sensitivity to broader departures from linear-Gaussian dependence, but that broader sensitivity does not by itself distinguish nonlinear dynamics from non-Gaussian innovations or non-stationary contributions, nor does it provide bifrequency localization.

BiSCA addresses a narrower but more specific target, namely quadratic phase coupling. It provides bifrequency-resolved diagnostics and distinguishes frequency-flat bicoherence, consistent with non-Gaussian input, from peaked bicoherence, consistent with quadratic coupling. Its scope is therefore complementary rather than universal, because it is designed for quadratic interactions and does not by itself test arbitrary higher-order nonlinear structure.

References

1. Frauscher, B. *et al.* Atlas of the normal intracranial electroencephalogram: neurophysiological awake activity in different cortical areas. *Brain* **141**, 1130–1144 (2018).
2. Li, M. *et al.* Harmonized-Multinational qEEG Norms (HarMNqEEG). *NeuroImage* 119190 (2022) doi:10.1016/j.neuroimage.2022.119190.
3. Travert, A. & Fernandez, C. SpectroChemPy. Zenodo https://doi.org/10.5281/ZENODO.3823841 (2024).
4. Lackey, H. E. *et al.* Practical guide to chemometric analysis of optical spectroscopic data. *J. Chem. Educ.* **100**, 2608–2626 (2023).
5. Price-Whelan, A. M., Lim, P. L., Earl, N., & Others. The Astropy Project: Sustaining and Growing a Community-oriented Open-source Project and the Latest Major Release (v5.0) of the Core Package. *Astrophys, J.* **935**, 167 (2022).
6. Guimarães, D. A. Segmented Regression via the Shape Language Modeling for Multi-Slope Path-Loss Modeling. in *Anais do XXXIX Simpósio Brasileiro de Telecomunicações e Processamento de Sinais* (Sociedade Brasileira de Telecomunicações, 2021). doi:10.14209/sbrt.2021.1570730012.

7. Newville, M., Stensitzki, T., Allen, D. B. & Ingargiola, A. LMFIT: Non-Linear Least-Square Minimization and Curve-Fitting for Python. Zenodo https://doi.org/10.5281/zenodo.11813 (2014).

8. Zetterberg, L. H. Estimation of parameters for a linear difference equation with application to EEG analysis. *Mathematical Biosciences* **5**, 227–275 (1969).

9. Isaksson, A., Lagergren, K. & Wennberg, A. Visible and non-visible EEG changes demonstrated by spectral parameter analysis. *Electroencephalography and Clinical Neurophysiology* **41**, 225–236 (1976).

10. Narasimhan, S. V. Pole-zero spectral modeling of EEG. *Signal Process.* **18**, 17–32 (1989).

11. Pascual-marqui, R. D., Valdes-sosa, P. A. & Alvarez-amador, A. A Parametric Model for Multichannel EEG Spectra. *International Journal of Neuroscience* **40**, 89–99 (1987).

12. Hughes, A. M., Whitten, T. A., Caplan, J. B. & Dickson, C. T. BOSC: A better oscillation detection method, extracts both sustained and transient rhythms from rat hippocampal recordings. *Hippocampus* **22**, 1417–1428 (2012).

13. Kosciessa, J. Q., Grandy, T. H., Garrett, D. D. & Werkle-Bergner, M. Single-trial characterization of neural rhythms: Potential and challenges. *NeuroImage* **206**, 116331 (2020).

14. Seymour, R. A., Alexander, N. & Maguire, E. A. Robust estimation of 1/f activity improves oscillatory burst detection. *European Journal of Neuroscience* **56**, 5836–5852 (2022).

15. Van Albada, S. & Robinson, P. Relationships between Electroencephalographic Spectral Peaks Across Frequency Bands. *Frontiers in Human Neuroscience* **7**, (2013).

16. Wen, H. & Liu, Z. Separating Fractal and Oscillatory Components in the Power Spectrum of Neurophysiological Signal. *Brain Topogr* **29**, 13–26 (2016).

17. Donoghue, T. *et al.* Parameterizing neural power spectra into periodic and aperiodic components. *Nature Neuroscience* **23**, 1655–1665 (2020).

18. Wilson, L. E., da Silva Castanheira, J. & Baillet, S. Time-resolved parameterization of aperiodic and periodic brain activity. *eLife* **11**, e77348 (2022).

19. Barry, R. J. & Blasio, F. M. D. Characterizing pink and white noise in the human electroencephalogram. *J. Neural Eng.* **18**, 034001 (2021).

20. Pascual-Marqui, R. D., Kochi, K. & Kinoshita, T. Cortical Xi-Alpha model for resting state electric neuronal activity. Preprint at https://doi.org/10.48550/arXiv.2212.13571 (2022).

21. Hu, S., Zhang, Z., Zhang, X., Wu, X. & Valdes-Sosa, P. A. \bm\xi-\bm\pi: a nonparametric model for neural power spectra decomposition. *IEEE J. Biomed. Health Inform.* **28**, 2624–2635 (2024).

22. Reyes, R. G. *et al.* Lifespan mapping of EEG source spectral dynamics with $\xi - \alpha$NET. 2025.02.21.639413 Preprint at https://doi.org/10.1101/2025.02.21.639413 (2025).

23. Hosking, J. R. M. & Wallis, J. R. *Regional Frequency Analysis: An Approach Based on L-Moments*. (Cambridge University Press, Cambridge, 1997). doi:10.1017/CBO9780511529443.

24. Valdés-Sosa, P. *et al.* The statistical identification of nonlinear brain dynamics: A progress report. in *Non linear Dynamic and Brain Funcioning* 278–284 (1999).

25. Nikias, C. & Petropulu, A. *Higher Order Spectra Analysis: A Non-Linear Signal Processing Framework*. (Pearson, Englewood Cliffs, N.J, 1993).

26. Zhou, R., Yu, Y. & Li, C. Revealing neural dynamical structure of C. elegans with deep learning. *Iscience* **27**, (2024).

27. Stiefel, K. M. & Ermentrout, G. B. Neurons as oscillators. *J. Neurophysiol.* **116**, 2950–2960 (2016).

28. Breakspear, M. Dynamic models of large-scale brain activity. *Nat. Neurosci.* **20**, 340–352 (2017).

29. Cole, S. R. & Voytek, B. Brain Oscillations and the Importance of Waveform Shape. *Trends Cogn Sci* **21**, 137–149 (2017).

30. Bartz, S., Avarvand, F. S., Leicht, G. & Nolte, G. Analyzing the waveshape of brain oscillations with bicoherence. *NeuroImage* **188**, 145–160 (2019).

31. Bender, A., Voytek, B. & Schaworonkow, N. Resting-state is not enough: alpha and mu rhythms change shape across development, but lack diagnostic sensitivity. 2023.10.13.562301 Preprint at https://doi.org/10.1101/2023.10.13.562301 (2023).

32. Schaworonkow, N. Overcoming harmonic hurdles: Genuine beta-band rhythms vs. contributions of alpha-band waveform shape. *Imaging Neuroscience* **1**, 1–8 (2023).

33. Shahbazi Avarvand, F. *et al.* Localizing bicoherence from EEG and MEG. *NeuroImage* **174**, 352–363 (2018).

34. Schaworonkow, N. & Nikulin, V. V. Is sensor space analysis good enough? Spatial patterns as a tool for assessing spatial mixing of EEG/MEG rhythms. *NeuroImage* **253**, 119093 (2022).

35. Shahbazi, F., Ewald, A. & Nolte, G. Univariate normalization of bispectrum using Hölder's inequality. *J Neurosci Methods* **233**, 177–186 (2014).

36. Evertz, R., Hicks, D. G. & Liley, D. T. J. Alpha blocking and 1/fβ spectral scaling in resting EEG can be accounted for by a sum of damped alpha band oscillatory processes. *PLOS Computational Biology* **18**, e1010012 (2022).

37. Lindén, H., Pettersen, K. H. & Einevoll, G. T. Intrinsic dendritic filtering gives low-pass power spectra of local field potentials. *J Comput Neurosci* **29**, 423–444 (2010).

38. Bédard, C., Kröger, H. & Destexhe, A. Does the $1/f$ Frequency Scaling of Brain Signals Reflect Self-Organized Critical States? *Phys. Rev. Lett.* **97**, 118102 (2006).

39. Valdes, P. A., Jimenez, J. C., Riera, J., Biscay, R. & Ozaki, T. Nonlinear EEG analysis based on a neural mass model. *Biological Cybernetics* **81**, 415–424 (1999).

40. Schetzen, M. *The Volterra and Wiener Theories of Nonlinear Systems*. (Wiley, 1980).

41. Marzocca, P., Nichols, J. M., Milanese, A., Seaver, M. & Trickey, S. Second-order spectra for quadratic nonlinear systems by Volterra functional series: Analytical description and numerical simulation. *Mechanical Systems and Signal Processing* **22**, 1882–1895 (2008).

42. Brillinger, D. R. An Introduction to Polyspectra. *The Annals of Mathematical Statistics* **36**, 1351–1374 (1965).
43. Brillinger, D. R. & Rosenblatt, M. ASYMPTOTIC THEORY OF ESTIMATES OF kTH-ORDER SPECTRA*. *Proceedings of the National Academy of Sciences* **57**, 206–210 (1967).
44. Komatsu, E. & Spergel, D. N. Acoustic signatures in the primary microwave background bispectrum. *Phys. Rev. D* **63**, 063002 (2001).
45. Brillinger, D. R. *Time Series: Data Analysis and Theory*. (SIAM: Society for Industrial and Applied Mathematics, 2001).
46. Berg, A., Paparoditis, E. & Politis, D. N. A bootstrap test for time series linearity. *Journal of Statistical Planning and Inference* **140**, 3841–3857 (2010).
47. Silva, L. R., Amitai, Y. & Connors, B. W. Intrinsic oscillations of neocortex generated by layer 5 pyramidal neurons. *Science* **251**, 432–435 (1991).
48. Van Kerkoerle, T. *et al.* Alpha and gamma oscillations characterize feedback and feedforward processing in monkey visual cortex. *Proc. Natl. Acad. Sci.* **111**, 14332–14341 (2014).
49. Mendoza-Halliday, D. *et al. A Ubiquitous Spectrolaminar Motif of Local Field Potential Power across the Primate Cortex*. http://biorxiv.org/lookup/doi/10.1101/2022.09.30.510398 (2022) doi:10.1101/2022.09.30.510398.
50. Brake, N. *et al.* A neurophysiological basis for aperiodic EEG and the background spectral trend. *Nat Commun* **15**, (2024).
51. Kopčanová, M. Resting-state EEG signatures of Alzheimer's disease are driven by periodic but not aperiodic changes. *Neurobiol. Dis.* 1–16 (2024) doi:10.1016/j.nbd.2023.106380.
52. Gallo, D. Differential effects of haloperidol on neural oscillations during wakefulness and sleep. 1–10 (2024).

53. Li, M. *et al.* Aperiodic and Periodic EEG Component Lifespan Trajectories: Monotonic Decrease versus Growth-then-Decline. 2025.08.26.672407 Preprint at https://doi.org/10.1101/2025.08.26.672407 (2025).

54. Gerster, M. *et al.* Separating Neural Oscillations from Aperiodic 1/f Activity: Challenges and Recommendations. *Neuroinform* **20**, 991–1012 (2022).

55. Nozari, E. *et al.* Macroscopic resting-state brain dynamics are best described by linear models. *Nat. Biomed. Eng.* **8**, 68–84 (2024).

56. Raffaelli, G. T., Jiříček, S. & Hlinka, J. Nonlinear brain connectivity from neurons to networks: quantification, sources and localization. 2024.11.17.623635 Preprint at https://doi.org/10.1101/2024.11.17.623635 (2024).
