# Supplementary material for: The influence of nonlinear resonance on human cortical oscillations
Source: Commun Biol. 2026 May 4;9:605. doi: 10.1038/s42003-026-10164-5 (PMC13144324; doi:10.1038/s42003-026-10164-5)
Supplement: Supplementary file 3 — Reporting Summary [file 42003_2026_10164_MOESM3_ESM.pdf]

nature portfolio

Corresponding author(s): Pedro A. Valdes-Sosa

Last updated by author(s): Nov 30, 2025

Reporting Summary

Nature Portfolio wishes to improve the reproducibility of the work that we publish. This form provides structure for consistency and transparency in reporting. For further information on Nature Portfolio policies, see our Editorial Policies and the Editorial Policy Checklist.

Statistics

For all statistical analyses, confirm that the following items are present in the figure legend, table legend, main text, or Methods section.

n/a	Confirmed	
☐	☒	The exact sample size (*n*) for each experimental group/condition, given as a discrete number and unit of measurement
☐	☒	A statement on whether measurements were taken from distinct samples or whether the same sample was measured repeatedly
☐	☒	The statistical test(s) used AND whether they are one- or two-sided<br>*Only common tests should be described solely by name; describe more complex techniques in the Methods section.*
☐	☒	A description of all covariates tested
☐	☒	A description of any assumptions or corrections, such as tests of normality and adjustment for multiple comparisons
☐	☒	A full description of the statistical parameters including central tendency (e.g. means) or other basic estimates (e.g. regression coefficient) AND variation (e.g. standard deviation) or associated estimates of uncertainty (e.g. confidence intervals)
☐	☒	For null hypothesis testing, the test statistic (e.g. *F*, *t*, *r*) with confidence intervals, effect sizes, degrees of freedom and *P* value noted<br>*Give P values as exact values whenever suitable.*
☒	☐	For Bayesian analysis, information on the choice of priors and Markov chain Monte Carlo settings
☒	☐	For hierarchical and complex designs, identification of the appropriate level for tests and full reporting of outcomes
☐	☒	Estimates of effect sizes (e.g. Cohen's *d*, Pearson's *r*), indicating how they were calculated

Our web collection on statistics for biologists contains articles on many of the points above.

Software and code

Policy information about availability of computer code

Data collection	No custom software was used for data collection. The study analyzed pre-existing datasets (HarMNqEEG and MNI Open iEEG Atlas).
Data analysis	The analysis code and derived data used in this study are publicly available on GitHub at https://github.com/rigelfalcon/BiSCA.

For manuscripts utilizing custom algorithms or software that are central to the research but not yet described in published literature, software must be made available to editors and reviewers. We strongly encourage code deposition in a community repository (e.g. GitHub). See the Nature Portfolio guidelines for submitting code & software for further information.

Data

Policy information about availability of data

All manuscripts must include a data availability statement. This statement should provide the following information, where applicable:

- Accession codes, unique identifiers, or web links for publicly available datasets
- A description of any restrictions on data availability
- For clinical datasets or third party data, please ensure that the statement adheres to our policy

The scalp EEG data are available from the HarMNqEEG dataset (Li et al., 2022; https://www.synapse.org/Synapse:syn26712693/wiki/). The intracranial EEG data are available from the MNI Open iEEG Atlas (Frauscher et al., 2018; https://ieegatlas.loris.ca/). The derived data supporting the findings of this study are available on GitHub at https://github.com/rigelfalcon/BiSCA.

Research involving human participants, their data, or biological material

Policy information about studies with human participants or human data. See also policy information about sex, gender (identity/presentation), and sexual orientation and race, ethnicity and racism.

Reporting on sex and gender	Sex/gender information is available in the demographic metadata of the cited datasets (HarMNqEEG and MNI Atlas). This study involved secondary analysis of existing data; sex and gender were not the primary variables of interest for the signal processing validation, but the datasets include both sexes.
Reporting on race, ethnicity, or other socially relevant groupings	This study utilized previously collected, anonymized multinational datasets. Race and ethnicity data were not analyzed as covariates in this signal processing study.
Population characteristics	The scalp EEG dataset consists of 960 healthy participants. The iEEG dataset consists of 106 patients with refractory focal epilepsy undergoing presurgical evaluation. Crucially, the recordings analyzed were strictly selected from non-lesional, non-epileptogenic tissue to approximate healthy activity (as validated in the original MNI Atlas publication, Frauscher et al., 2018).
Recruitment	We did not recruit participants directly. The study analyzes pre-existing, publicly available datasets. Recruitment procedures for the original datasets are detailed in Li et al., 2022 (HarMNqEEG) and Frauscher et al., 2018 (MNI Atlas).
Ethics oversight	Ethical approval was not required for this specific study as it exclusively analyzed pre-existing, publicly available, and anonymized datasets. The collection of the original data received ethical approval from the respective local authorities as detailed in the original publications.

Note that full information on the approval of the study protocol must also be provided in the manuscript.

Field-specific reporting

Please select the one below that is the best fit for your research. If you are not sure, read the appropriate sections before making your selection.

☒ Life sciences ☐ Behavioural & social sciences ☐ Ecological, evolutionary & environmental sciences

For a reference copy of the document with all sections, see nature.com/documents/nr-reporting-summary-flat.pdf

Life sciences study design

All studies must disclose on these points even when the disclosure is negative.

Sample size	We analyzed resting-state EEG data from 960 healthy participants from the HarMNqEEG dataset. For intracranial EEG (iEEG), the final dataset included 105 patients (1,771 electrodes) from the MNI iEEG atlas after applying the exclusion criteria detailed below. No statistical methods were used to predetermine sample size; we utilized all available high-quality recordings from these established open datasets to maximize statistical power.
Data exclusions	For scalp EEG, recordings shorter than 1 minute were excluded. Data were segmented into 1.5s epochs, and segments were retained only if artifact-free. For iEEG, we utilized the curated MNI Open iEEG Atlas; the original providers of this dataset had already excluded channels from lesional zones, seizure onset zones, or those showing interictal discharges. In the current study, we further excluded patients who had only a single valid channel remaining, because the inverse solution required for amplitude scale correction (using the IOLMM model described in the manuscript) cannot be performed on single-channel recordings.
Replication	To ensure computational reproducibility, the complete BiSCA analysis framework, including source code and demonstration examples, has been open-sourced on GitHub (https://github.com/rigelfalcon/BiSCA). As the study utilizes public datasets, all results can be fully reproduced using the provided codebase. Scientifically, the findings were successfully replicated across two independent, large-scale modalities (scalp EEG and intracranial EEG), which showed consistent patterns of nonlinearity. In-silico simulations were also performed to validate the method's reliability. All attempts at replication were successful.
Randomization	Randomization was not relevant to this study because it is an observational analysis of existing resting-state electrophysiological data. There were no experimental intervention groups.
Blinding	Blinding was not relevant because the study involved the computational analysis of retrospective, anonymized datasets without group allocation or treatment arms.

Reporting for specific materials, systems and methods

We require information from authors about some types of materials, experimental systems and methods used in many studies. Here, indicate whether each material, system or method listed is relevant to your study. If you are not sure if a list item applies to your research, read the appropriate section before selecting a response.

Materials & experimental systems

n/a	Involved in the study
☒	☐ Antibodies
☒	☐ Eukaryotic cell lines
☒	☐ Palaeontology and archaeology
☒	☐ Animals and other organisms
☒	☐ Clinical data
☒	☐ Dual use research of concern
☒	☐ Plants

Methods

n/a	Involved in the study
☒	☐ ChIP-seq
☒	☐ Flow cytometry
☒	☐ MRI-based neuroimaging

Plants

Seed stocks	*Report on the source of all seed stocks or other plant material used. If applicable, state the seed stock centre and catalogue number. If plant specimens were collected from the field, describe the collection location, date and sampling procedures.*
Novel plant genotypes	*Describe the methods by which all novel plant genotypes were produced. This includes those generated by transgenic approaches, gene editing, chemical/radiation-based mutagenesis and hybridization. For transgenic lines, describe the transformation method, the number of independent lines analyzed and the generation upon which experiments were performed. For gene-edited lines, describe the editor used, the endogenous sequence targeted for editing, the targeting guide RNA sequence (if applicable) and how the editor was applied.*
Authentication	*Describe any authentication procedures for each seed stock used or novel genotype generated. Describe any experiments used to assess the effect of a mutation and, where applicable, how potential secondary effects (e.g. second site T-DNA insertions, mosiacism, off-target gene editing) were examined.*
